# Conjugation-Modulated Excitonic Coupling Brightens Multiple Triplet Excited States

**DOI:** 10.1021/jacs.2c12320

**Published:** 2023-01-13

**Authors:** Tao Wang, Abhishek Kumar Gupta, Sen Wu, Alexandra M. Z. Slawin, Eli Zysman-Colman

**Affiliations:** Organic Semiconductor Centre, EaStCHEM School of Chemistry, University of St Andrews, Andrews KY16 9ST, U.K.

## Abstract

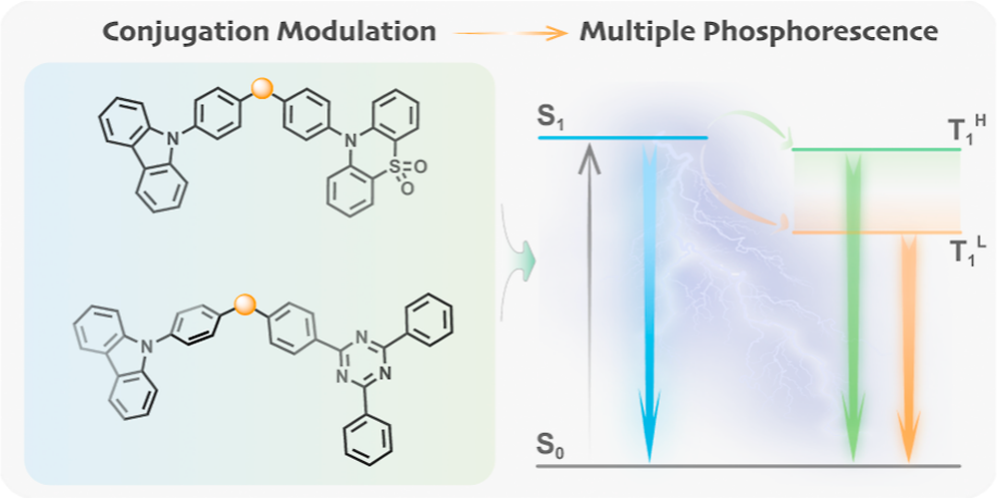

The design and regulation of multiple room-temperature phosphorescence (RTP) processes are formidably challenging due to the restrictions imposed by Kasha’s rule. Here, we report a general design principle for materials that show multiple RTP processes, which is informed by our study of four compounds where there is modulation of the linker hybridization between donor (D) and acceptor (A) groups. Theoretical modeling and photophysical experiments demonstrate that multiple RTP processes can be achieved in sp^3^ C-linked D–A compounds due to the arrest of intramolecular electronic communication between two triplet states (T_1_^H^ and T_1_^L^) localized on the donor and acceptor or between two triplet states, one localized on the donor and one delocalized across aggregated acceptors. However, for the sp^2^ C-linked D–A counterparts, RTP from one locally excited T_1_ state is observed because of enhanced excitonic coupling between the two triplet states of molecular subunits. Single-crystal and reduced density gradient analyses reveal the influence of molecular packing on the coincident phosphorescence processes and the origin of the observed aggregate phosphorescence. These findings provide insights into higher-lying triplet excited-state dynamics and into a fundamental design principle for designing compounds that show multiple RTP.

## Introduction

The efficient generation, controllable management, and on-demand migration of triplet excitons from higher-lying to lower-lying triplet excited states in organic molecules have combined to constitute the long-sought Holy Grail of room-temperature phosphorescence (RTP). However, the exciton spin-flip between well-separated singlet and triplet excited states is not allowed due to the violation of Winger’s rule.^1,2^ To tackle this apparent contradiction, ever-increasingly complex molecular design principles have been devised to modulate both the molecular structure and host–guest interactions.^3−5^ Behind these is the control of the relative kinetics of facilitating intersystem crossing (ISC)^6,7^ and suppressing nonradiative processes,^8−10^ both of which are paramount to achieving efficient RTP. From the perspective of molecular design, introducing heteroatoms, such as N,^11,12^ O,^13,14^ P,^15−17^ and S,^18−20^ into metal/halogen-free aromatic compounds is a popular way to enhance the ISC efficiency through the introduction of *n*–π* states that allow for the spin-flip process to occur, following El-Sayed’s rule.^21^ Most recently, Marder and co-workers demonstrated that a (σ, B_p_) → (π, B_p_) transition localized on a three-coordinate boron atom could also accelerate ISC.^22^ This new approach likewise relies on El-Sayed’s rule to mediate state mixing and thus ISC from S_1_ to T_1_. To sufficiently brighten the populated triplet excitons of organic compounds, it is also necessary to weaken nonradiative energy dissipation. To date, a plethora of methods, such as engineering molecular crystals,^11,14,23−25^ employing a polymer matrix,^26−30^ and constructing a rigid host environment,^31−34^ have been exploited to achieve suppressed nonradiative decay. Following these principles, many RTP luminophores have been developed, showing moderate photoluminescence (PL) quantum yield (Φ_PL_) and/or long phosphorescence lifetime (τ_Ph_). These compounds have been exploited in diverse applications, such as imaging,^35−37^ optoelectronics,^38−40^ nonlinear optics,^41^ and information encryption.^16,18,28,42^

Multiple phosphorescence from a single component is an emergent phenomenon from the radiative decay of excitons from multiple accessible triplet excited states. Compounds that show multiple phosphorescence offer a handle into the study of triplet exciton dynamics. Due to Kasha’s rule,^43^ it remains a formidable challenge to enable multiple phosphorescence at room temperature, though there are well-studied systems that show anti-Kasha’s fluorescence.^44,45^ Thus, there have been only a few reports documenting anti-Kasha RTP in organic compounds.^46−48^ For example, Wu and co-workers reported that heterocyclic stilbene derivatives exhibited room-temperature dual phosphorescence originating from both T_2_ and T_1_ states.^47^ This results from the relatively large energy gap (>0.5 eV) between these two states, which inhibits the excitonic internal conversion (IC). Room-temperature dual phosphorescence has also been demonstrated in both systems where there is simultaneous emission from monomers and aggregates.^11,49^ Recently, T_1_ conformer-regulated multiple phosphorescence has been demonstrated by us, which renders the excited-state dynamics tunable via thermal activation.^20,50^ However, a general principle to regulate multiple phosphorescence on demand remains elusive.

**Figure 1:**
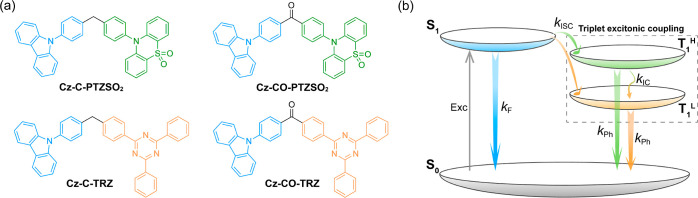
(a) Chemical structures investigated in this study. (b) Schematic illustration of a Jablonski diagram outlining the origin of multiple phosphorescence from T_1_^H^ and T_1_^L^ states. Exc: Excitation; F: fluorescence; Ph: phosphorescence; ISC: intersystem crossing; IC: internal conversion.

Here, we present a general multiple RTP design strategy for organic compounds by modulating the excitonic coupling between two T_1_ states localized on the donor and acceptor via the degree of conjugation that the linker mediates between these two groups. We define the higher-lying and lower-lying T_1_ states as T_1_^H^ and T_1_^L^ states, respectively. By comparing the photophysics of emitters containing sp^3^ and sp^2^ linkages (C vs CO), we found that dual phosphorescence relies on how strongly the two triplet excited states communicate. It was demonstrated in two examples that sp^3^ C-linked compounds (**Cz-C-PTZSO**_**2**_ and **Cz-C-TRZ**) (Figure 1a), where 9-phenylcarbazole (**Cz-Ph**) serves as the donor and 10-phenyl-10*H*-phenothiazine-5,5-dioxide (**PTZSO**_**2**_) or 2,4,6-triphenyl-1,3,5-triazine (**TRZ**) acts as the acceptor (Scheme S1a), could enable dual phosphorescence from two locally excited (^3^LE) triplet states due to negligible excitonic coupling between donor and acceptor moieties at room temperature (Figure 1b). In contrast, the sp^2^ C-linked counterpart (**Cz-CO-PTZSO**_**2**_) shows only one ^3^LE emission at room temperature, together with aggregate phosphorescence in the crystal state, while no RTP was recorded in the other sp^2^ C-linked compound (**Cz-CO-TRZ**).

## Results and Discussion

The synthesis of **Cz-C-PTZSO**_**2**_, **Cz-CO-PTZSO**_**2**_, **Cz-C-TRZ**, and **Cz-CO-TRZ** is outlined in Scheme S1. All samples were purified by repeated silica column chromatography, followed by temperature-gradient vacuum sublimation and finally recrystallization from toluene. The molecular structures and purity were validated by a combination of ^1^H and ^13^C nuclear magnetic resonance (NMR) spectroscopy, high-resolution mass spectrometry (HRMS), melting point determination, elemental analysis (EA), and high-performance liquid chromatography (HPLC) (Figures S1–S32).

We hypothesized that the IC between donor- and acceptor-localized T_1_ states could be slowed or even inhibited through the use of a nonconjugated sp^3^ carbon atom to electronically isolate donor and acceptor groups (**Cz-C-PTZSO**_**2**_ and **Cz-C-TRZ**), where multiple RTP would originate from both donor and acceptor subunits (Figure 1b). For sp^2^ C-linked species (**Cz-CO-PTZSO**_**2**_ and **Cz-CO-TRZ**), due to the stronger electronic communication between the donor and acceptor, the IC of excitons between the two low-lying triplet states is greatly enhanced, resulting in only phosphorescence from the T_1_^L^ state.

**Figure 2:**
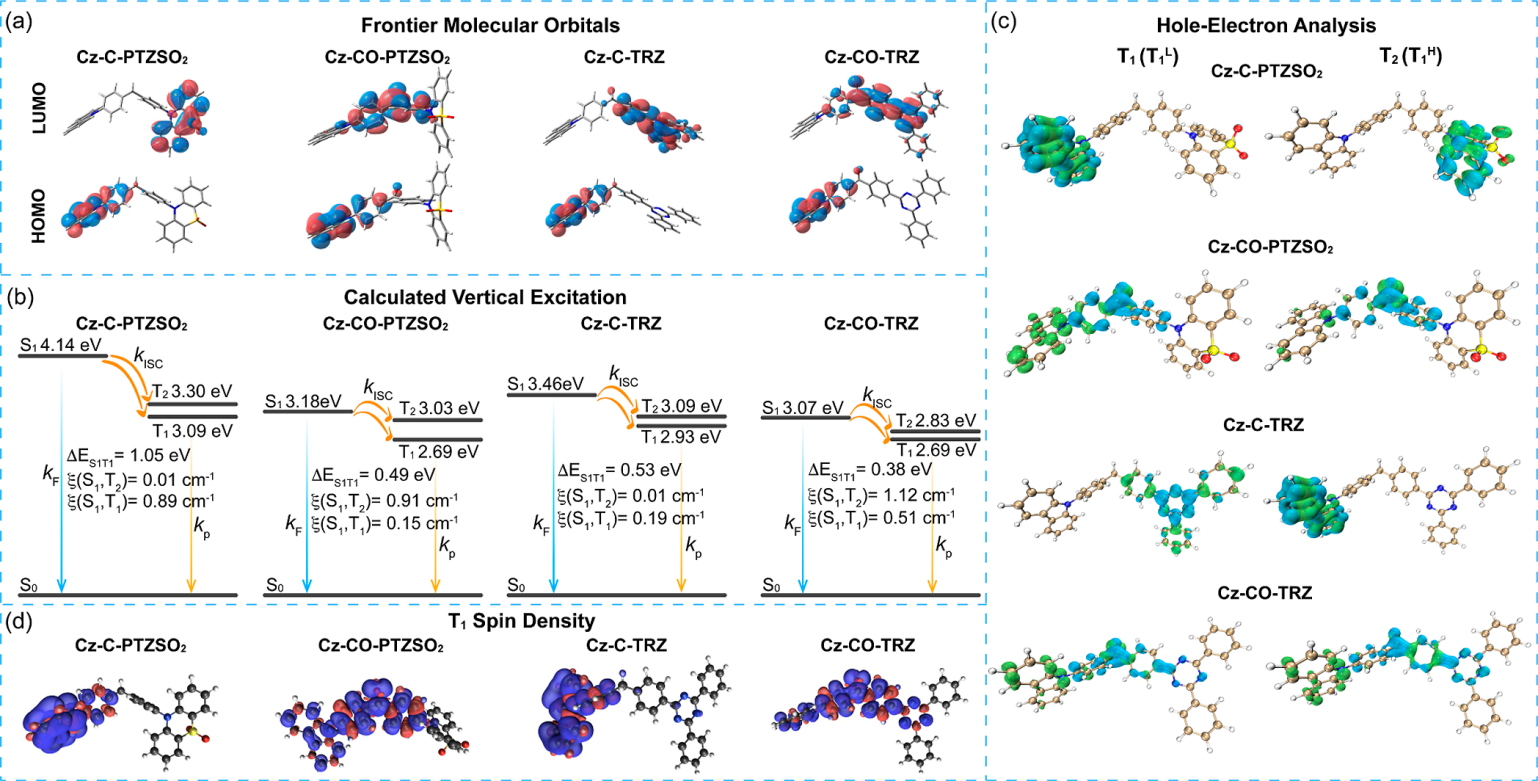
(a) Electron density distribution of the frontier molecular orbitals (isovalue: 0.02) and (b) vertical excitation energy levels of S_1_, T_1_, and T_2_ states calculated at the optimized S_0_ geometry in the gas phase at the TD-DFT-PBE0/6-31G(d,p) level; spin–orbit coupling constants calculated at the S_1_ geometry. (c) Hole (green)–electron (blue) distribution analysis (isovalue: 0.002) for triplet excited states at the optimized S_0_ geometry. (d) T_1_ spin density distributions (isovalue: 0.0004) calculated at the optimized T_1_ geometry in the gas phase at the uPBE0/6-31G(d,p) level.

To substantiate this hypothesis, we first modeled the photophysical properties of these four compounds in the gas phase using density functional theory (DFT) at the PBE0^51^/6-31G(d,p) level.^52^ From Figure 2a, the highest occupied molecular orbital (HOMO) and lowest unoccupied molecular orbital (LUMO) of **Cz-C-PTZSO**_**2**_ and **Cz-C-TRZ** are localized on the donor (**Cz-Ph**) and acceptors (**PTZSO**_**2**_ and **TRZ**), showing a near-zero frontier molecular orbital (FMO) overlap. For **Cz-CO-PTZSO**_**2**_ and **Cz-CO-TRZ**, the LUMO is delocalized across both the acceptor and the benzophenone core, resulting in deeper HOMO and LUMO levels (Figure S33). Time-dependent DFT calculations at the same level of theory demonstrate a relatively large energy gap between S_1_ and T_1_ states (ΔE_ST_) in these compounds (Figure 2b), indicating that these compounds should not exhibit thermally activated delayed fluorescence. Given the presence of multiple triplet state energy levels serving as upper receiver states and moderate spin–orbital coupling constants (Figure S34), RTP should be theoretically favored as the dominant radiative decay process. The T_1_ and T_2_ energies of **Cz-C-PTZSO**_**2**_ and **Cz-C-TRZ** are almost degenerated with those of the T_1_ energies of the isolated donor, **Cz-Ph**, and acceptors, **PTZSO**_**2**_ and **TRZ** (Figure S35b). For **Cz-CO-PTZSO**_**2**_ and **Cz-CO-TRZ**, similar T_1_ energies imply that both states have the same character, which is close to that of the LE T_1_ state of the carbazole-benzophenone (**Cz-BP**) subunit (Figure S35b). Furthermore, the excitonic coupling between T_1_ and T_2_ was investigated based on the hole–electron distribution analysis using the Multiwfn program.^53^**Cz-C-PTZSO**_**2**_ and **Cz-C-TRZ** both show decoupled T_1_ and T_2_ states (Figure 2c), where the hole (green) and electron (blue) distributions are identical to those of the donor (as the T_1_ state for **Cz-C-PTZSO**_2_; as the T_2_ state for **Cz-C-TRZ**) and the acceptor (as the T_2_ state for **Cz-C-PTZSO**_2_; as the T_1_ state for **Cz-C-TRZ**) (Figure S35c). Considering that the T_1_ and T_2_ states are localized on the donor and acceptor subunits and possess almost identical triplet energies with the T_1_ energies of the isolated subunits, here we redefine the T_2_ and T_1_ states as T_1_^H^ and T_1_^L^ states, respectively. According to Fermi’s golden rule,^54^ the reduced vibronic coupling between T_1_^H^ and T_1_^L^ states should also favor a slow IC, enabling emission from both the donor and acceptor groups. However, **Cz-CO-PTZSO**_**2**_ and **Cz-CO-TRZ** exhibit strongly coupled triplet excited states due to the overlap of the hole and electron densities as well as the small energy gap between T_1_^H^ and T_1_^L^ states; thus, there should be rapid IC between T_1_^H^ and T_1_^L^ states. T_1_ spin density distributions reveal that in **Cz-C-PTZSO**_**2**_ and **Cz-C-TRZ**, they are localized on **Cz-Ph** (Figure 2d), while for **Cz-CO-PTZSO**_**2**_ and **Cz-CO-TRZ**, the spin densities are relatively delocalized (Figure 2d), similar to the hole–electron density distribution analysis (Figure 2c). Calculated T_1_ energies at the optimized T_1_ geometry are 2.86, 2.03, 2.86, and 1.98 eV for **Cz-C-PTZSO**_**2**_, **Cz-CO-PTZSO**_**2**_, **Cz-C-TRZ**, and **Cz-CO-TRZ**, respectively.

**Figure 3:**
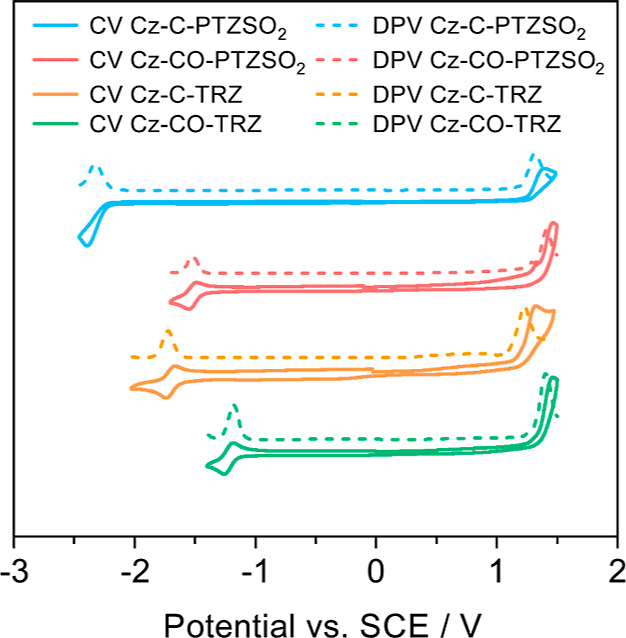
Cyclic voltammogram and differential pulse voltammogram in degassed DMF with 0.1 M [^*n*^Bu_4_N]PF_6_ as the supporting electrolyte and Fc/Fc^+^ as the internal reference (0.45 V vs SCE).^55^*E*_HOMO/LUMO_ = −(*E*^ox^/*E*^red^ + 4.8) eV using values vs Fc/Fc^+^.^56^

The energies of FMOs were inferred from the oxidation and reduction potentials (*E*^ox^/*E*^red^), obtained using cyclic voltammetry and differential pulse voltammetry (DPV) in deaerated DMF with 0.1 M [^*n*^Bu_4_N]PF_6_ as the supporting electrolyte. The *E*^ox^/*E*^red^ values of **Cz-C-PTZSO**_**2**_, **Cz-CO-PTZSO**_**2**_, **Cz-C-TRZ**, and **Cz-CO-TRZ**, determined from the DPV peaks, are 1.31/–2.32 V, 1.42/–1.52 V, 1.23/–1.73 V, and 1.41/–1.18 V, respectively, versus SCE (Figure 3, Table S1). The corresponding HOMO/LUMO values are −5.66/–2.03, −5.77/–2.83, −5.57/–2.62, and −5.76/–3.17 eV,^55^ respectively. The more stabilized HOMO values of **Cz-CO-PTZSO**_**2**_ and **Cz-CO-TRZ** compared with those of **Cz-C-PTZSO**_**2**_ and **Cz-C-TRZ** are the result of the presence of the electron-withdrawing benzophenone fragment that stabilizes the HOMO. Very similar HOMO values were calculated for **Cz-C-PTZSO**_**2**_ and **Cz-C-TRZ**, reflecting the electronically isolated donor in both these compounds (Figure 2a), which is absent in **Cz-CO-PTZSO**_**2**_ and **Cz-CO-TRZ**. The trends in measured HOMO/LUMO levels coincide with those modeled using DFT (Figure S33).

**Figure 4:**
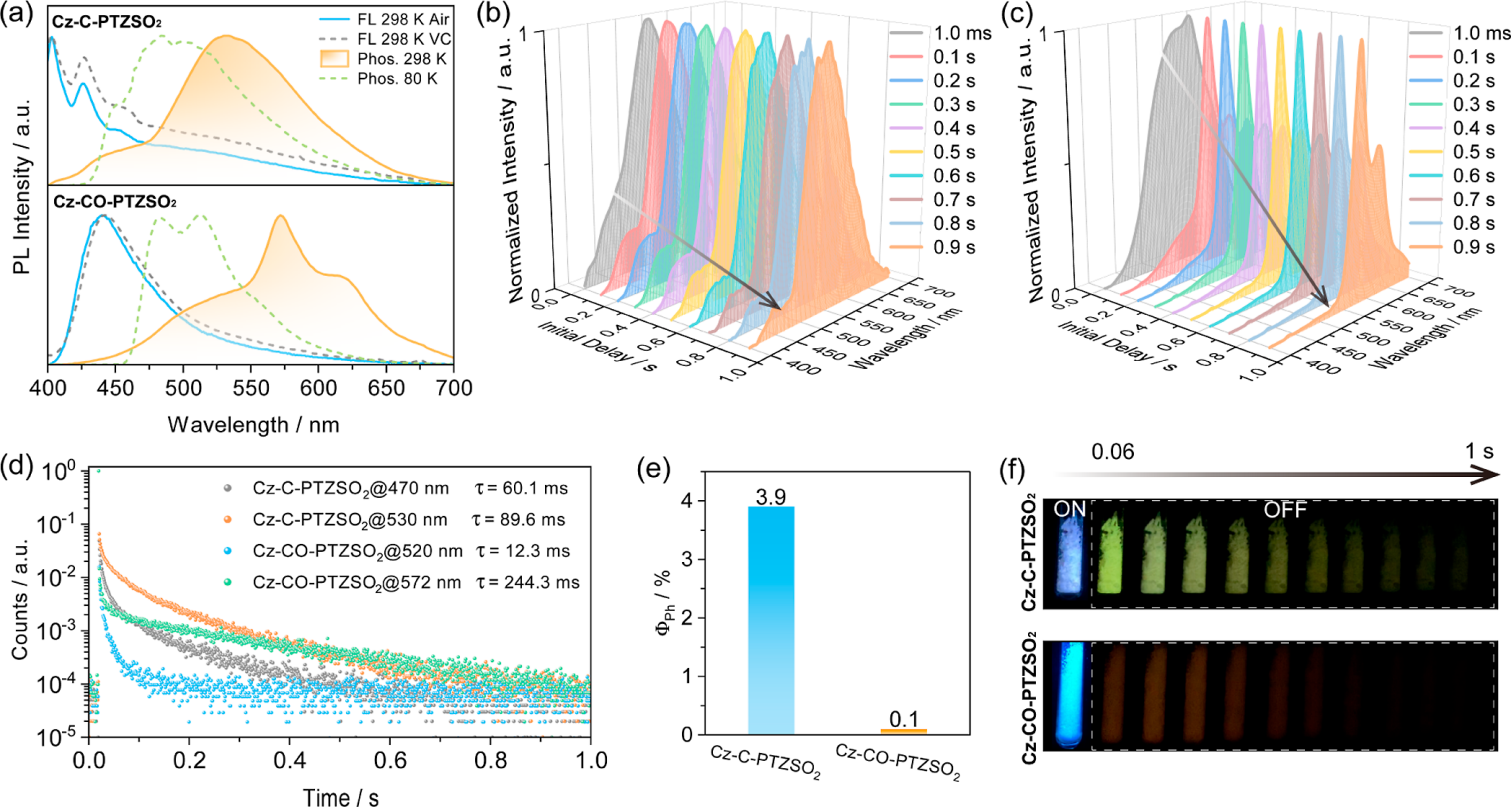
(a) Steady-state PL (in air and in vacuum) and phosphorescence spectra (in vacuum); λ_exc_ = 370 nm; time-gated window: 3 ms to 1 s (**Cz-C-PTZSO**_**2**_), 3–200 ms for 298 K and 3 ms to 1 s for 80 K (**Cz-CO-PTZSO**_**2**_); VC denotes vacuum. RTP spectra of (b) **Cz-C-PTZSO**_**2**_ and (c) **Cz-CO-PTZSO**_**2**_ in vacuum recorded at different delay times and a gate of 100 ms. (d) Time-resolved phosphorescence decay profiles in vacuum at 298 K. (e) RTP Φ_PL_ values in N_2_ at 298 K. (f) Images showing phosphorescence afterglow in vacuum at 298 K (excitation source: 365 nm UV torch).

We next investigated the photophysical properties of the **Cz-C-PTZSO**_**2**_ and **Cz-CO-PTZSO**_**2**_ crystals, which were isolated from a toluene solution. The photophysical data are summarized in Table S2. The steady-state PL spectrum of **Cz-C-PTZSO**_**2**_ in air at 298 K shows a structured LE emission profile with peak maxima, λ_PL_, at 403 and 426 nm, alongside a broad emission tail (Figure 4a). The corresponding PL lifetime, τ_PL_, is ∼3.5 ns, recorded at both 403 and 426 nm (Figure S36a). When placed under vacuum, the broad PL band was enhanced with λ_PL_ at 403 and 426 nm, indicating the most likely involvement of triplet excited states. Time-gated PL measurements (3 ms delay) detected the RTP spectrum of **Cz-C-PTZSO**_**2**_, where dual emission bands centered at 470 and 530 nm can be observed (Figure 4a), together with associated average phosphorescence lifetime, τ_Ph_, values of 60.1 and 89.6 ms (Figure 4d), respectively. Figure 4b shows RTP spectra collected at different delay times, which demonstrate that the RTP at 470 nm decays faster than that at 530 nm, reflecting the results of the RTP lifetime measurements (Figure 4d). These dual emission bands match well with the enhanced LE emission and the emission tail presented in the steady-state PL spectra in vacuum. The phosphorescence spectrum recorded at 80 K blue-shifts to 475 nm and is dominated by a structured blue emission band, whose low-temperature phosphorescence (LTP) lifetime is prolonged to 836.7 ms and longer than that recorded at 530 nm (τ_Ph_ = 536.6 ms) (Figure S37a). In line with the proposed multiple phosphorescence model (Figure 1b), we ascribe the dual RTP emission to result from phosphorescence from each of the **Cz-Ph** and **PTZSO**_**2**_ moieties. To verify these assignments, we measured the phosphorescence spectra of **Cz-Ph** and **PTZSO**_**2**_ at 77 K (Figures S38 and S39a). It was found that one of the two RTP emission bands of **Cz-C-PTZSO**_**2**_ matches the phosphorescence of the **Cz-Ph** monomers (Figure S38), while the low-energy RTP band is red-shifted compared to the phosphorescence of **PTZSO**_**2**_ (Figure S39a); rather, this phosphorescence aligns with the phosphorescence of **PTZSO**_**2**_ aggregates (Figure S39a). Time-gated phosphorescence measurements of **Cz-C-PTZSO**_**2**_ recorded in dilute 2-MeTHF glass at 77 K reveal the evolving structured phosphorescence spectra as a function of time (Figure S40a). Distinct from the phosphorescence behavior observed in the crystal, in 2-MeTHF glass, dual phosphorescence is observed originating from LE phosphorescence of the **Cz-Ph** donor (T_1_^L^) and **PTZSO**_**2**_ acceptor (T_1_^H^), which aligns with the DFT calculations.

The behavior of **Cz-CO-PTZSO**_**2**_ is different. The steady-state PL spectra in air and vacuum at 298 K are similar (λ_PL_ = 440 nm) (Figure 4a), with a PL lifetime of 0.7 ns (Figure S36b). Although dual RTP behavior was recorded, it differs from that of **Cz-C-PTZSO**_**2**_. The blue RTP emission band from **Cz-Ph** is absent in **Cz-CO-PTZSO**_**2**_, but a new structured RTP emission band is present at λ_PL_ of 572 nm. At 80 K, the phosphorescence emission blue-shifts to the green emission range, whose low-temperature LTP lifetime is 89.8 ms (Figure S37b). We assigned dual phosphorescence to originate from dual emission from the monomer and the aggregates, which is consistent with the recorded PL spectra of the model systems. The computed hole–electron distribution analysis revealed that the phosphorescence of **Cz-CO-PTZSO**_**2**_ results from LE T_1_ emission from **Cz-BP** (Figure 2c). Therefore, we measured the phosphorescence spectrum of **Cz-BP** at 77 K (Figure S39b), which coincides with the green RTP emission band of **Cz-CO-PTZSO**_**2**_. We can therefore assign this band to phosphorescence from the **Cz-BP** moiety. To determine the origin of the orange RTP emission, we collected RTP spectra at various delay times. The results revealed that the RTP at 520 nm decays much faster than the RTP at 572 nm (Figure 4c), with corresponding τ_Ph_ of 12.3 ms and 244.3 ms, respectively (Figure 4d). By closer inspection of the phosphorescence spectra of dilute samples in 2-MeTHF glass at 77 K, we found that the emission centered at 572 nm vanished (Figure S41b), implying that the lower-energy RTP of **Cz-CO-PTZSO**_**2**_ crystals originates from aggregates. As a result, we can conclude that dual phosphorescence of **Cz-CO-PTZSO**_**2**_ belongs to the mixture of T_1_ emission of the monomer and aggregates rather than T_1_^H^ and T_1_^L^ emissions from different subunits. The Φ_PL_ values of **Cz-C-PTZSO**_**2**_ and **Cz-CO-PTZSO**_**2**_ at 298 K in air are 5.9 and 4.6%, respectively, which improve to 8.2 and 4.7% under a N_2_ atmosphere, respectively (Table S2). Therefore, the calculated RTP Φ_PL_ values of **Cz-C-PTZSO**_**2**_ and **Cz-CO-PTZSO**_**2**_ in N_2_ are approximately 4% and 0.1%, respectively (Figure 4e). The weaker RTP of **Cz-CO-PTZSO**_**2**_ may be caused by the presence of increased nonradiative channels. Figure 4f shows the RTP afterglows of **Cz-C-PTZSO**_**2**_ and **Cz-CO-PTZSO**_**2**_ in vacuum.

**Figure 5:**
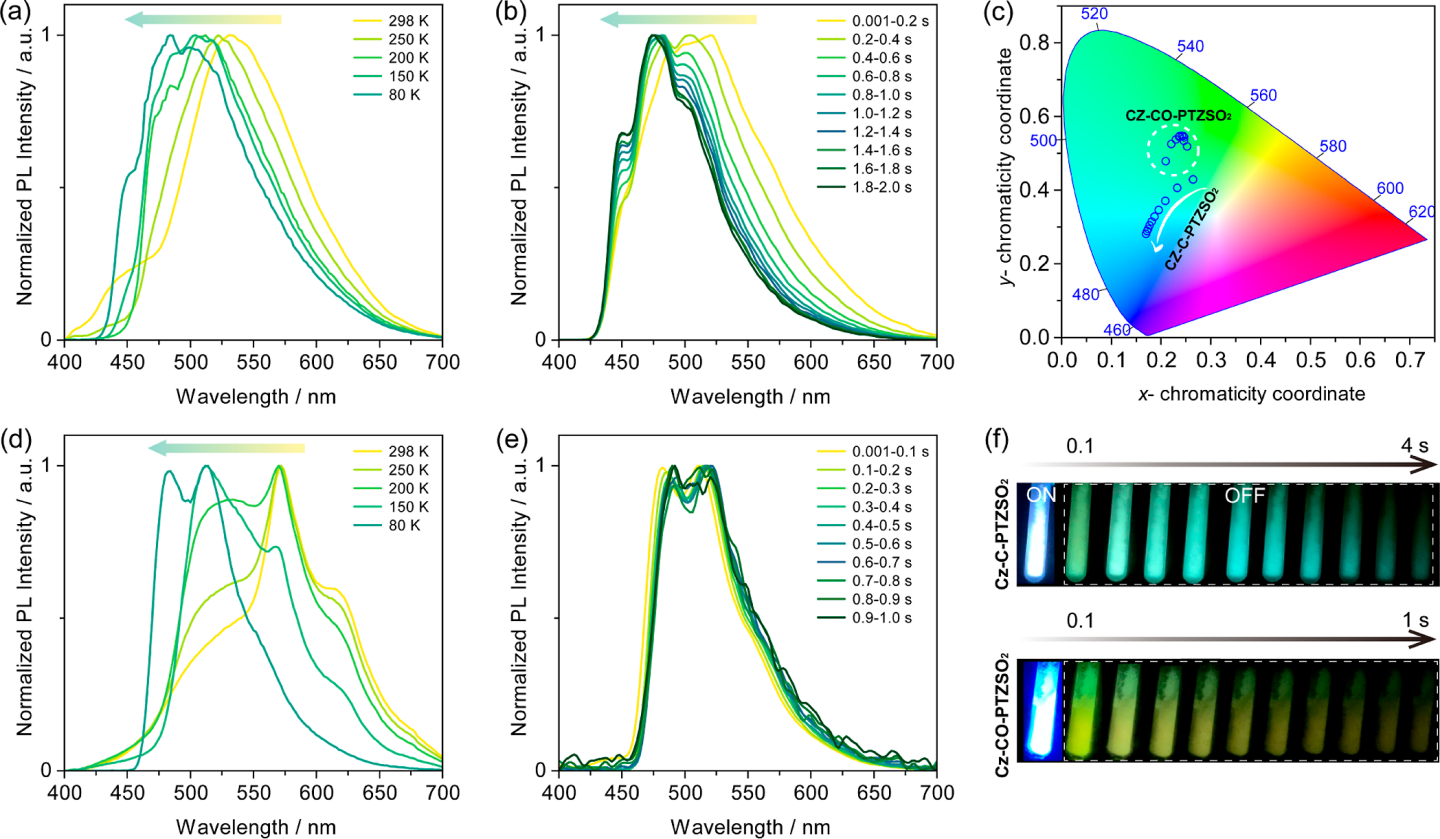
Temperature-dependent phosphorescence spectra of (a) **Cz-C-PTZSO**_**2**_ (time-gated window: 3 ms to 1 s) and (d) **Cz-CO-PTZSO**_**2**_ (time-gated window: 3–200 ms for 298–150 K; 3 ms to 1 s for 80 K); λ_exc_ = 370 nm. Phosphorescence of (b) **Cz-C-PTZSO**_**2**_ and (e) **Cz-CO-PTZSO**_**2**_ at 80 K recorded at various time-gated windows. (c) CIE coordinates of phosphorescence at 80 K of **Cz-C-PTZSO**_**2**_ and **Cz-CO-PTZSO**_**2**_ as a function of time-gated windows shown in (b,e). (f) Images showing phosphorescence afterglows in vacuum at 77 K (excitation source: 365 nm UV torch).

Further, temperature-dependent PL measurements were conducted to interrogate the origin of the multiple phosphorescence. For **Cz-C-PTZSO**_**2**_, the apparent blue shift of the phosphorescence emission occurs as the temperature decreases (Figure 5a), indicating that phosphorescence from **Cz-Ph** gradually dominates the spectral profile at lower temperatures. At 80 K, blue-shifted and structured phosphorescence spectra at different time-gated windows were observed, which confirmed that phosphorescence from T_1_^H^ and T_1_^L^ states of **Cz-C-PTZSO**_**2**_ is dominated by the LE emission from the **Cz-Ph** and **PTZSO**_**2**_ groups (Figure 5b), respectively, as these spectra align with those of the phosphorescence spectra of **Cz-Ph** monomers and **PTZSO**_**2**_ aggregates (Figures S38 and S39a); the T_1_^H^ and T_1_^L^ energies, estimated from the emission peak maxima, are 2.36 and 2.64 eV, respectively. The related Commission Internationale de l′Éclairage (CIE) diagram reflects the gradual emission color change across different time-gated windows (Figure 5c). The images of **Cz-C-PTZSO**_**2**_ also evidence that the afterglow gradually varies from green to blue (Figure 5f). The approximated calculated slow IC rate between T_1_^H^ and T_1_^L^ indicates that there is weak communication between the two triplet excited states associated with the donor and acceptor subunits (Table S3), which explains the occurrence of T_1_^H^ phosphorescence. A similar scenario was observed in **Cz-CO-PTZSO**_**2**_, where temperature-dependent phosphorescence blue-shifts as a function of decreasing temperature (Figure 5d). However, at 80 K, phosphorescence from **Cz-BP** dominates the emission, independent of the time-gated window (Figure 5e). The CIE diagram and images of afterglows document the unchanged phosphorescence color (Figure 5c,f) over time, indicating that the emission results from phosphorescence originating from the **Cz-BP** moiety of **Cz-CO-PTZSO**_**2**_ (i.e., from the T_1_ state). The ΔE_ST_ of **Cz-C-PTZSO**_**2**_ and **Cz-CO-PTZSO**_**2**_ in dilute samples in 2-MeTHF glass at 77 K, estimated from the difference in energy between the onsets of the prompt and delayed emission spectra, are 0.28 and 0.41 eV, respectively (Figure S41); it should be noted that ΔE_ST_ of **Cz-C-PTZSO**_**2**_ is in fact the energy gap between S_1_ and T_1_^H^ states (**Cz-Ph**).

**Figure 6:**
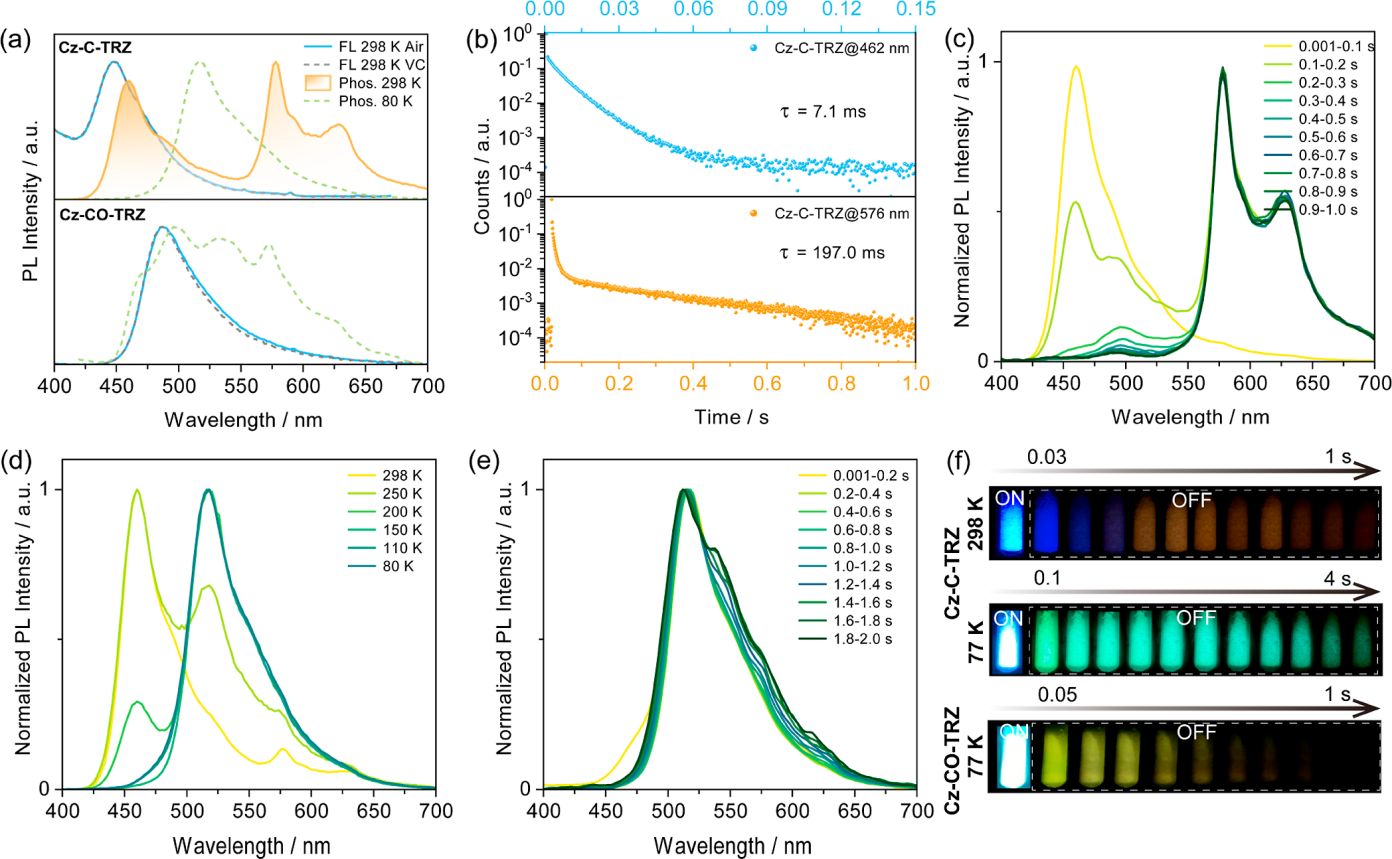
(a) Steady-state PL (in air and in vacuum) and phosphorescence spectra (in vacuum); λ_exc_ = 370 nm; time-gated window: 0.05–1 s for 298 K and 0.05–2 s for 80 K (**Cz-C-TRZ**), 0.05–0.8 s for 80 K for **Cz-CO-TRZ**. (b) Time-resolved phosphorescence decay profiles of **Cz-C-TRZ** in vacuum at 298 K. Phosphorescence spectra of **Cz-C-TRZ** in vacuum at (c) 298 and (e) 80 K recorded at different delay times and a gate of 100 ms. (d) Temperature-dependent phosphorescence spectra of **Cz-C-TRZ**; time-gated window: 3 ms to 1 s. (f) Images showing phosphorescence afterglows in vacuum (excitation source: 365 nm UV torch).

Next, the photophysical properties of the **Cz-C-TRZ** and **Cz-CO-TRZ** crystals were investigated. There is no obvious change to the steady-state PL spectra of **Cz-C-TRZ** in air and vacuum, centered at 448 nm (Figure 6a), whose PL lifetime is 5.1 ns measured at 298 K in air (Figure S42a). At 298 K, time-gated PL measurements detected two well-separated phosphorescence bands centered at 460 and 575 nm, with associated lifetimes of 7.1 and 197 ms, respectively (Figure 6b). However, at 80 K, only one green phosphorescence band at 515 nm (τ_Ph_ = 400 ms, Figure S43) was observed (Figure 6a). Compared to the phosphorescence spectra of each of **Cz-Ph** and **TRZ** (Figures S38 and S39c), we conclude that blue RTP and green LTP originate from states localized on **Cz-Ph** and **TRZ**, denoted as T_1_^H^ and T_1_^L^, respectively, similar to the theoretical prediction (Figure 2c). Similarly, variation of the relative intensity of the structured phosphorescence spectrum of **Cz-C-TRZ** in 2-MeTHF glass recorded at 77 K at different time-gated windows also evidences contributions from the emission from both the T_1_^H^ and T_1_^L^ states associated with the **Cz-Ph** and **TRZ** moieties, respectively (Figure S40b). Given that the **TRZ** moiety is prone to forming π–π stacked assemblies, we postulated that the orange RTP likely originates from molecular aggregates. To better elucidate the origin of the multiple triplet excited states, phosphorescence spectra were recorded at different time-gated windows. As shown in Figure 6c, at early time, blue RTP is dominant. Then, emission from aggregates dominates the overall RTP emission, accompanied by a small contribution from the **TRZ** phosphorescence at around 500 nm. The RTP afterglow color of **Cz-C-TRZ** varies from blue to orange (Figure 6f). Temperature-dependent phosphorescence measurements also documented that multiple emissive triplet states exist in the **Cz-C-TRZ** crystals (Figure 6d). At 80 K, LTP emission negligibly changes with time (Figure 6e), reflected by the observed unchanged LTP afterglow (Figure 6f). To further confirm that the orange RTP is from aggregates, the RTP emission of **Cz-C-TRZ** crystals after grinding was recorded. We found that the overall RTP intensity was weakened, where the blue RTP intensity ratio was increased compared to the orange RTP due to the disrupted intermolecular interactions in the aggregates (Figure S44). We prepared **Cz-C-TRZ** crystals via slow sublimation, which we hypothesized would produce samples with increased π–π stacking. These samples of **Cz-C-TRZ** show stronger orange RTP and negligible blue RTP (Figure S45a,b,e). The RTP lifetime and RTP Φ_PL_ of **Cz-C-TRZ** crystals that were acquired via temperature-gradient vacuum sublimation increase to 540.3 ms and 3.0%, respectively (Figure S46a, Table S4) compared to the respective values of the **Cz-C-TRZ** crystals (τ_Ph_ = 190.7 ms, RTP Φ_PL_ = 0.6%) isolated from toluene. At 80 K, green LTP from **TRZ** centered at 500 nm was also observed, likely due to the suppression of nonradiative processes (Figure S45c), whose lifetime is prolonged to 820.8 ms (Figure S46b). For **Cz-CO-TRZ**, steady-state PL spectra show an unstructured emission centered at 486 nm at room temperature (Figure 6a), whose overall Φ_PL_ and lifetime in air are 13.9% and 2.4 ns, respectively (Table S2, Figure S42b). Unfortunately, no RTP was observed, probably resulting from the strong nonradiative decay. A broad and structured LTP profile was recorded at 80 K (Figure 6a). Time-gated PL measurements reflect that the LTP results from LE emission contributions from each of **Cz-Ph**, **Cz-BP**, and **TRZ** (Figure S47) based on the cross-comparison with the LTP from each of these isolated compounds (Figures S38 and S39b,c). The low-temperature multiple phosphorescence bands may result from restricted molecular conformations that lead to weak electronic coupling at low temperature.^20,57^

**Figure 7:**
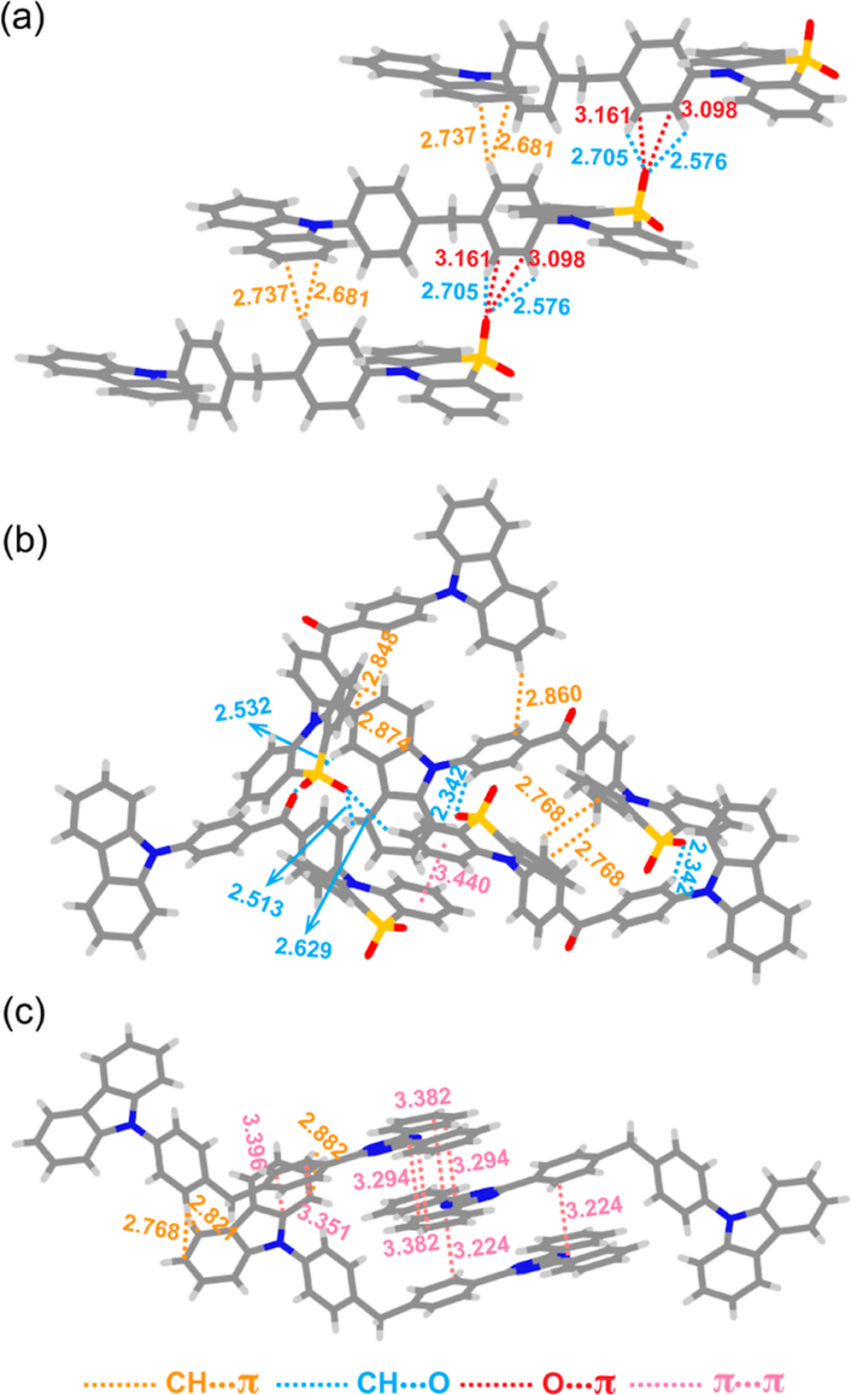
Single-crystal structures of (a) **Cz-C-PTZSO**_**2**_, (b) **Cz-CO-PTZSO**_**2**_, and (c) **Cz-C-TRZ** showing intermolecular interactions. The distance unit is Å.

We then investigated the single-crystal structures of **Cz-C-PTZSO**_**2**_, **Cz-CO-PTZSO**_**2**_, and **Cz-C-TRZ** to determine what influence if any intermolecular interactions would have on the observed multiple phosphorescence. For **Cz-C-PTZSO**_**2**_, strong CH···O and O···π intermolecular interactions exist between **PTZSO**_**2**_ moieties (Figure 7a), which is consistent with the assignment that the T_1_^L^ emission of **Cz-C-PTZSO**_**2**_ at room temperature is almost identical to that from the acceptor **PTZSO**_**2**_ aggregates. No intermolecular interactions were found between the **Cz-Ph** fragments, implying that the emission from the T_1_^H^ state is from monomeric **Cz-Ph**. For **Cz-CO-PTZSO**_**2**_, intermolecular CH···π, CH···O, and π···π interactions are present (Figure 7b), which is consistent with the orange RTP of **Cz-CO-PTZSO**_**2**_ originating from **Cz-CO-PTZSO**_**2**_ aggregates, in addition to the green RTP from the **Cz-BP** moiety. For **Cz-C-TRZ**, π···π intermolecular interactions exist between adjacent TRZ fragments (Figure 7c), which result in excitonic splitting and thus a lower T_1_ energy (orange RTP). Similar to **Cz-C-PTZSO**_**2**_, no apparent intermolecular interactions exist between the adjacent **Cz-Ph** groups, resulting in the occurrence of RTP from **Cz-Ph** in **Cz-C-TRZ**.

**Figure 8:**
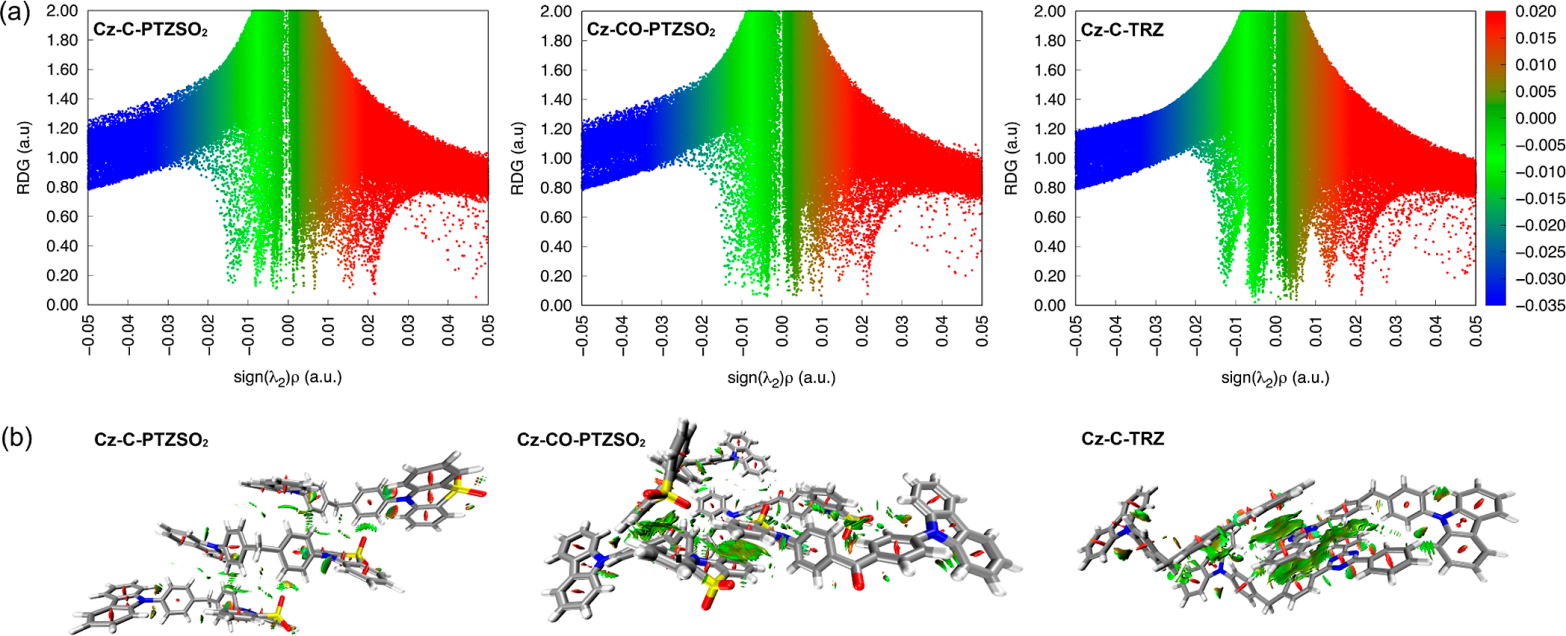
(a) Plots of RDG vs the electron density (ρ) multiplied by the sign of the second Hessian eigenvalue (λ_2_). (b) RDG mapped with isosurfaces (isovalue: 0.6) showing intermolecular interactions.

In addition, intermolecular interactions were studied in silico by employing noncovalent interaction calculations^58^ implemented in the Multiwfn program.^53^ The geometries of the emitters are those in the single crystals. As shown in Figure 8a, green spikes of the reduced density gradient (RDG), where the electron density is near zero, confirm the existence of intense van der Waals interactions, which can suppress molecular motions resulting in reduced nonradiative decay in the solid state. The RDG isosurfaces of **Cz-C-PTZSO**_**2**_ reveal noncovalent interactions between the **PTZSO**_**2**_ groups (Figure 8b) and negligible interactions between **Cz-Ph** groups, similar to the conclusions drawn from the single-crystal analysis. For **Cz-CO-PTZSO**_**2**_, evident noncovalent interactions are distributed across the entirety of the molecules, implicating that the orange RTP belongs to **Cz-CO-PTZSO**_**2**_ aggregates. As anticipated, π–π interactions are present in the **TRZ** moiety of **Cz-C-TRZ** and CH···π and π····π interactions exist between **Cz-Ph** and **TRZ**. As a result, molecular packing of **Cz-C-TRZ** influences the occurrence of dual RTP, where condensed molecular packing results in strong **Cz-Ph** and **TRZ** interactions and thus efficient triplet–triplet energy transfer (TTET); that is, only orange RTP can be observed in this instance.

## Conclusions

Herein, we have reported a general multiple RTP design principle and validated it using four organic compounds (**Cz-C-PTZSO**_**2**_, **Cz-CO-PTZSO**_**2**_, **Cz-C-TRZ**, and **Cz-CO-TRZ**). DFT calculations demonstrated that for sp^3^ C-linked compounds, dual RTP could be enabled from both the donor and acceptor fragments due to the absence of excitonic coupling between the T_1_^H^ and T_1_^L^ triplet excited states. However, the excitonic coupling channel of the two states is enabled by employing an sp^2^ C as a linker between the donor and acceptor moieties, resulting in only one ^3^LE emission. Photophysical investigations substantiated this design principle. For **Cz-C-PTZSO**_**2**_ and **Cz-C-TRZ** crystals, dual RTP emissions from T_1_^H^ and T_1_^L^ states were recorded, which are LE emissions from the donor and acceptor fragments, respectively, together with the acceptor aggregate phosphorescence. For the **Cz-CO-PTZSO**_**2**_ crystal, only one LE T_1_-dominated RTP was observed due to the strong excitonic coupling between the donor and acceptor; benefitting from the multiple intermolecular interactions, molecular aggregate phosphorescence was also activated. However, for **Cz-CO-TRZ**, there is no RTP, probably due to significant nonradiative decay. Single-crystal and RDG analyses demonstrated that noncovalent interactions influence multiple RTP behavior and TTET. This work provides insights for the design of organic systems that show dual phosphorescence and discloses an effective strategy for designing color-variable RTP that can potentially be exploited in a range of applications.

## Data Availability

The research data supporting this publication can be accessed at https://doi.org/10.17630/773b2b1c-dfc8-4967-be09-fbd0c8b2199e.

## Supporting Information Available

^1^H and ^13^C NMR spectra, HRMS, HPLC, and EA of the target compounds and supplementary computational and photophysical data (PDF)
Crystallographic data for **Cz-C-PTZSO**_**2**_, **Cz-CO-PTZSO**_**2**_, and **Cz-C-TRZ** (XYZ)

## Supplementary Material

ja2c12320_si_001.pdf

ja2c12320_si_002.xyz
