# Supplementary material for: Conjugation-Modulated Excitonic Coupling Brightens Multiple Triplet Excited States
Source: J Am Chem Soc. 2023 Jan 13;145(3):1945–54. doi: 10.1021/jacs.2c12320 (PMC9880999; doi:10.1021/jacs.2c12320)
Supplement: Supplementary file 1 — ja2c12320_si_001.pdf [file ja2c12320_si_001.pdf]

Supporting Information

Conjugation-Modulated Excitonic Coupling Brightens Multiple Triplet Excited States

*Tao Wang, Abhishek Kumar Gupta, Sen Wu, Alexandra M. Z. Slawin, Eli Zysman-Colman**

Organic Semiconductor Centre, EaStCHEM School of Chemistry, University of St Andrews, St Andrews KY16 9ST, UK.

** E-mail: eli.zysman-colman@st-andrews.ac.uk; http://www.zysman-colman.com*

Table of Contents

General Methods 3

Experimental Section 7

Photophysical Data 33

References 46

General Methods

General synthetic procedures

10*H*-phenothiazine 5,5-dioxide was synthesized according to the literature procedure.[1] Before use, carbazole was purified by silica gel column chromatography and further purified by recrystallization in methanol and vacuum sublimation. All other reagents and solvents were obtained from commercial sources and used as received. Air-sensitive reactions were performed under a nitrogen atmosphere using Schlenk techniques, and no special precautions were taken to exclude air or moisture during work-up. Flash column chromatography was carried out using silica gel (Silica-P from Silicycle, 60 Å, 40-63 μm). Analytical thin-layer-chromatography (TLC) was performed with silica plates with aluminum backings (250 μm with F-254 indicator). TLC visualization was accomplished by a 254/365 nm UV lamp. ^{1}H and ^{13}C NMR spectra were recorded on a Bruker Advance spectrometer (500 MHz for ^{1}H, 125 MHz for ^{13}C) in d_6-DMSO, $CDCl_3$ and CD_2Cl_2. The following abbreviations have been used for multiplicity assignments: “s” for singlet, “d” for doublet, “t” for triplet, “q” for quartet, “m” for multiplet. ^{1}H and ^{13}C NMR spectra were referenced residual solvent peaks with respect to TMS (δ = 0 ppm). Melting points were measured using open-ended capillaries on an Electrothermal 1101D Mel-Temp apparatus and are uncorrected. High-resolution mass spectrometry (HRMS) was performed at the University of Edinburgh. Elemental analyses were performed by the School of Geosciences at the University of Edinburgh.

X-Ray crystallography

Single Crystals of **Cz-C-PTZSO$_2$**, **Cz-CO-PTZSO$_2$**, and **Cz-C-TRZ** were isolated from organic solvents via a slow evaporation process. X-ray diffraction data were collected at 173 K using a Rigaku FR-X Ultrahigh brilliance Microfocus RA generator/confocal optics with XtaLAB P200 diffractometer [Mo Kα radiation (λ = 0.71075 Å)]. Intensity data were collected using ω-steps accumulating area detector images spanning at least a hemisphere of reciprocal space. Data were collected using CrystalClear[2, 3] and processed (including correction for Lorentz polarization and absorption) using CrysAlisPro.[4, 5] Structures were solved by dual-

space methods (SHELXT-2018/2) and refined by full-matrix least-squares against F^2 (SHELXL-2018/3).[6] Non-hydrogen atoms were refined anisotropically, and hydrogen atoms were refined using a riding model. All calculations were performed using the Olex2 interface.[6, 7] CCDC 2203534, 2203533, and 2203532 contain supplementary crystallographic data for this paper. The data can be obtained free of charge from The Cambridge Crystallographic Data Centre via www.ccdc.cam.ac.uk/structures.

Electrochemistry measurements

Cyclic Voltammetry (CV) analysis was performed on an Electrochemical Analyzer potentiostat model 620E from CH Instruments at a sweep rate of 100 mV/s. Differential pulse voltammetry (DPV) was conducted with an increment potential of 0.01 V and a pulse amplitude, width, and period of 50 mV, 0.06, and 0.5 s, respectively. Samples were prepared as *N, N*-dimethylformamide solutions, degassed by sparging with DMF-saturated N_2 for 5 minutes before measurements. All measurements were performed using 0.1 M DMF solution of tetra-*n*-butylammonium hexafluorophosphate ([nBu$_4$N]PF$_6$]). An Ag/Ag^+ electrode was used as the reference electrode, while a glassy carbon electrode and a platinum wire were used as the working electrode and counter electrode, respectively. The redox potentials are reported relative to a saturated calomel electrode (SCE) with a ferrocenium/ferrocene (Fc/Fc^+) redox couple as the internal standard (0.45 V vs. SCE).[8] The HOMO and LUMO energies were determined using the relation $E_{HOMO}/_{LUMO} = -(E_{ox} / E_{red} + 4.8)$ eV, where E_{ox} and E_{red} are the onset of anodic and cathodic peak potentials, respectively calculated from DPV relative to Fc/Fc^+.[9]

Theoretical calculations

All ground state optimizations have been carried out at the Density Functional Theory (DFT) level with Gaussian 16[10] using the PBE0 functional[11] and the 6-31G(d,p) basis set.[12] Vibrational frequency calculations were performed to ensure that the optimized geometries represented the local minima. Excited-state calculations have been performed at time-dependent DFT (TD-DFT) using the same functional and basis set as for ground state geometry optimization. Spin-

orbit coupling matrix elements (ξ) were calculated based on the optimized singlet excited state geometry. Spin-orbit coupling matrix elements between singlet and triplet excited states were calculated using the PySOC program.[13] Triplet excited state optimizations were conducted at the uPBE0/6-31G(d,p) level. Triplet emissions were calculated using TD-DFT at the same level of theory at the optimized T_1 geometry. Molecular orbitals were visualized using GaussView 6.0.[14] Hole-electron distribution and reduced density gradient (RDG)[15] analyses were conducted using the Multiwfn program,[16] and the corresponding molecular orbitals were visualized using VMD program.[17]

Photophysical measurements

Optically dilute solutions of concentrations on the order of 10^{-5} or 10^{-6} M were prepared in spectroscopic or HPLC grade solvents for absorption and emission analysis. Absorption spectra were recorded at room temperature on a Shimazu UV-2600 double beam spectrophotometer with a 1 cm quartz cuvette. Molar absorptivity determination was verified by linear regression analysis of values obtained from at least four independent solutions at varying concentrations with absorbance ranging from 8.0×10^{-6} to 1×10^{-5} M.

Steady-state and time-resolved emission spectra were recorded at 298 K using an Edinburgh Instruments FS5. Samples were excited at 370 nm for steady-state measurements and time-resolved measurements. Phosphorescence emission spectra were collected with a 5 W microsecond flash lamp by the multi-channel scaling (MCS) mode.

An integrating sphere (SC-30 module on FS5 fluorimeter) was employed for the photoluminescence quantum yield measurements of solid samples. The Φ_{PL} of the films were then measured in air and N_2 environment by purging the integrating sphere with N_2 gas flow.

The singlet-triplet energy splitting (ΔE_{ST}) in 2-MeTHF was estimated from the onset of prompt fluorescence spectra and phosphorescence emission at 77 K. The samples were excited by a femtosecond laser emitting at 343 nm (Orpheus-N, model: SP-06-200-PP). Emission from the samples was focused onto a spectrograph (Chromex imaging, 250is spectrograph) and detected on a sensitive gated iCCD camera (Stanford Computer Optics, 4Picos) having subnanosecond resolution. Phosphorescence spectra of 2-MeTHF glass were measured from 1

ms after photoexcitation, with an iCCD exposure time was 7.5 ms. Prompt fluorescence spectra were measured from 1 ns after photoexcitation with an iCCD exposure time was 99 ns.

Fitting of time-resolved luminescence measurements

Time-resolved PL measurements were fitted to a sum of exponential decay model, with chi-squared (χ^2) values between 1 and 2, using the EI FLS980 software. Each component of the decay is assigned a weight, (w_i), which is the contribution of the emission from each component to the total emission.

The average lifetime was then calculated using the following:

- Two exponential decay model:

$$\tau_{AVG} = \tau_1 w_1 + \tau_2 w_2$$

with weights defined as $w_1 = \frac{A1\tau_1}{A1\tau_1 + A2\tau_2}$ and $w_2 = \frac{A2\tau_2}{A1\tau_1 + A2\tau_2}$ where A1 and A2 are the preexponential-factors of each component.

- Three exponential decay model:

$$\tau_{AVG} = \tau_1 w_1 + \tau_2 w_2 + \tau_3 w_3$$

with weights defined as $w_1 = \frac{A1\tau_1}{A1\tau_1 + A2\tau_2 + A3\tau_3}$, $w_2 = \frac{A2\tau_2}{A1\tau_1 + A2\tau_2 + A3\tau_3}$ and $w_3 = \frac{A3\tau_3}{A1\tau_1 + A2\tau_2 + A3\tau_3}$ where A1, A2 and A3 are the preexponential-factors of each component.

Experimental Section

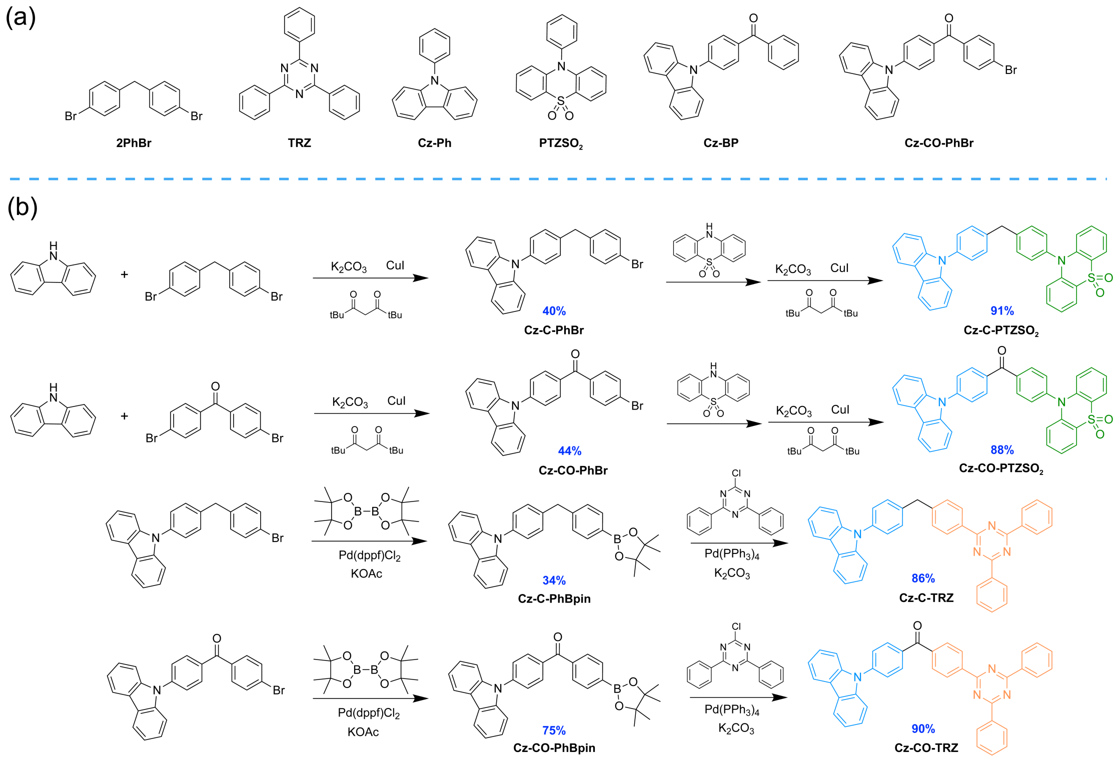

Scheme S1. (a) Reference and intermediate compounds. **2PhBr**,[18] **TRZ**,[19] **Cz-Ph**,[20] **PTZSO$_2$**,[21] **Cz-BP**,[22] and **Cz-CO-PhBr**[23] were synthesized according to the reported reference. (b) Synthetic routes of **Cz-C-PTZSO$_2$**, **Cz-CO-PTZSO$_2$**, **Cz-C-Trz**, and **Cz-CO-Trz**. The purity of carbazole has been verified by ^{1}H & ^{13}C NMR spectroscopy and HPLC (Figures **S1-S3**).

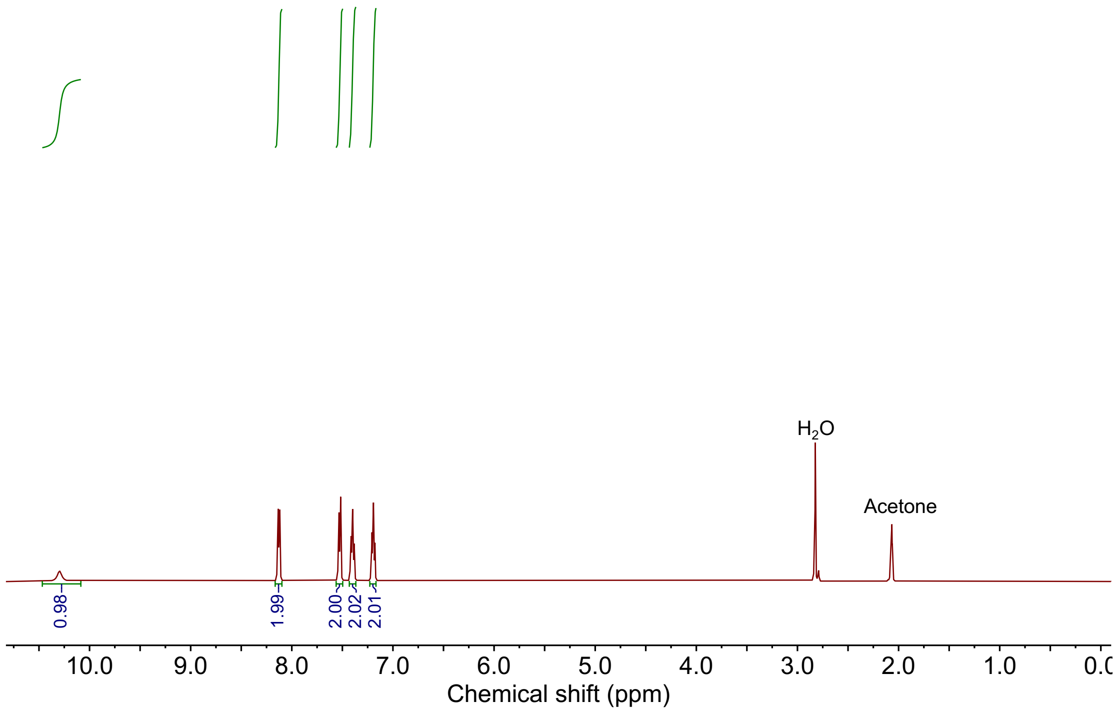

Figure S1. ^{1}H NMR spectrum of carbazole in d_6-acetone.

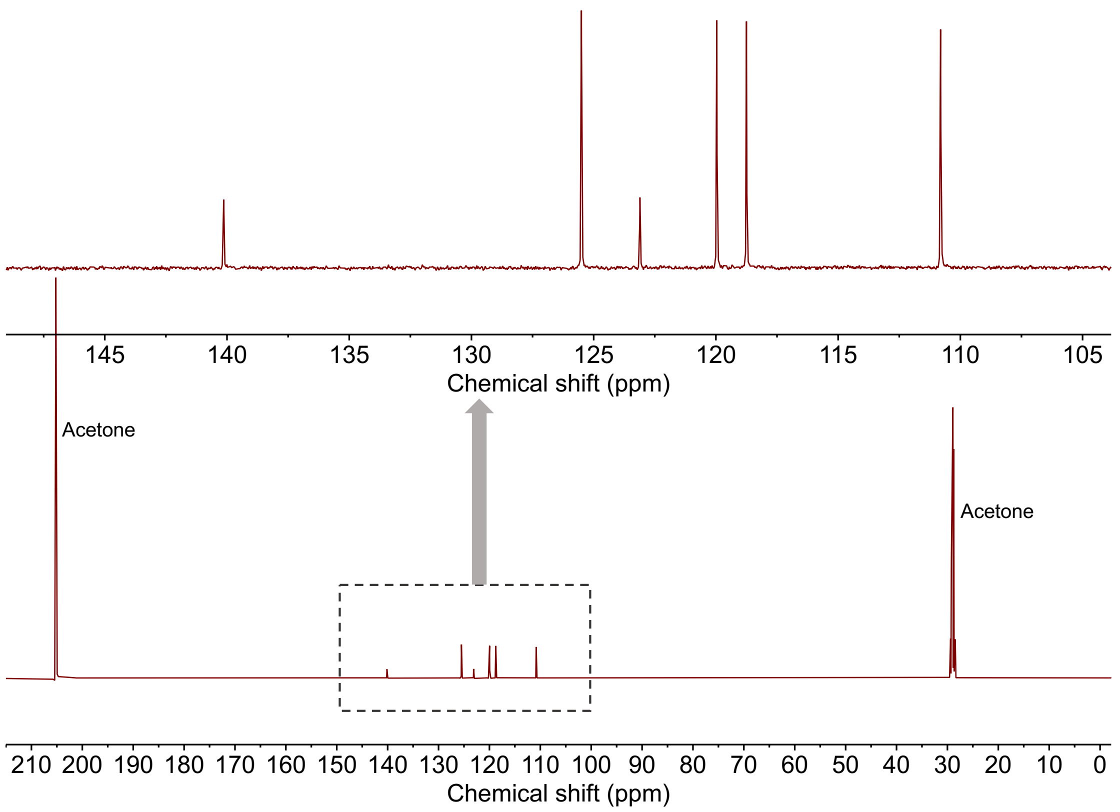

Figure S2. ^{13}C NMR spectrum of carbazole in d_6-acetone.

HPLC Trace Report29Aug2022

<Sample Information>

Sample Name	: cz8		
Sample ID	:		
Method Filename	: 55ac 45h2o 20min.lcm **Elution solvent: 55%MeCN/45%H2O**		
Batch Filename	: a.lcb		
Vial #	: 1-5	Sample Type	: Unknown
Injection Volume	: 10 uL		
Date Acquired	: 29/08/2022 15:17:38	Acquired by	: Qingzhi Zhang
Date Processed	: 29/08/2022 16:52:48	Processed by	: System Administrator

<Chromatogram>

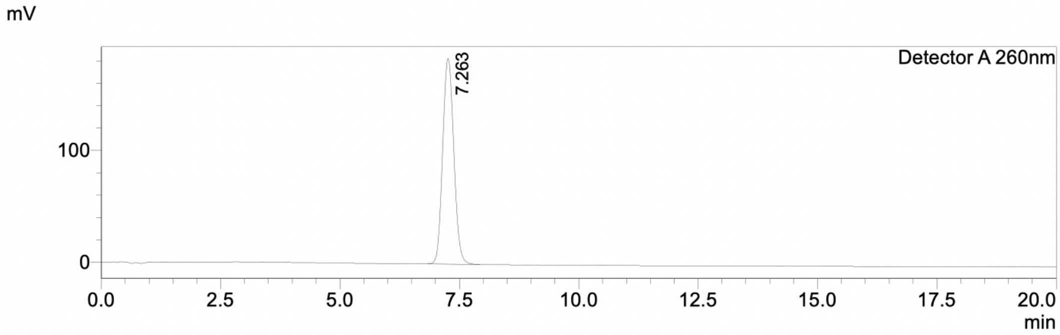

<Peak Table>

Detector A 260nm

Peak#	Ret. Time	Area	Height	Area%	Area/Height	Width at 5% Height
1	7.263	2969576	184219	100.000	16.120	0.527
Total		2969576	184219	100.000		

Figure S3. HPLC trace of carbazole.

Synthesis of 9-(4-(4-bromobenzyl)phenyl)-9H-carbazole (Cz-C-PhBr)

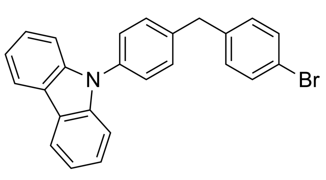

The synthetic route follows the Ullmann coupling reaction according to the literature.[24] Carbazole (2.00 g, 11.96 mmol, 1.0 equiv.), **2PhBr** (5.85 g, 17.94 mmol, 1.5 equiv.), potassium carbonate (3.31 g, 23.92 mmol, 2.0 equiv.), copper iodide (0.23 g, 1.20 mmol, 0.1 equiv.), and 2,2,6,6-tetramethylheptane-3,5-dione (0.44 g, 2.39 mmol, 0.2 equiv.) were added to a Schlenk flask containing 60 mL of anhydrous DMF. After degassing the flask, the reaction system was placed under a nitrogen atmosphere. The reactant mixture was heated at 150 °C for 24 h. After cooling to room temperature, DCM (200 mL) was added to the mixture. The organic solution was then washed with a saturated NaCl aqueous solution (200 mL × 2) and further with a 1 M

HCl solution (200 mL). The collected organic phase was dried over anhydrous magnesium sulfate and concentrated under reduced pressure. The collected crude product was purified by silica gel column chromatography (DCM: Hexane = 1:7, **R**$_f$: 0.24) to afford the desired compound as a white solid. **Yield**: 1.97 g, 40%. **Mp**: 114-115 °C. **^{1}H NMR (500 MHz, *d*$_6$-DMSO) δ (ppm)**: 8.27 – 8.20 (m, 2H), 7.58 – 7.47 (m, 6H), 7.42 (ddd, J = 8.2, 6.9, 1.3 Hz, 2H), 7.37 – 7.31 (m, 4H), 7.28 (td, J = 7.5, 1.1 Hz, 2H), 4.09 (s, 2H). **^{13}C NMR (126 MHz, CD_2Cl_2) δ (ppm)**: 140.88, 140.06, 140.04, 135.77, 131.61, 130.83, 130.24, 127.07, 125.90, 123.21, 120.18, 120.02, 119.83, 109.73, 40.89. **GC-MS** (m/z): 411.10; retention time: 14.762 min.

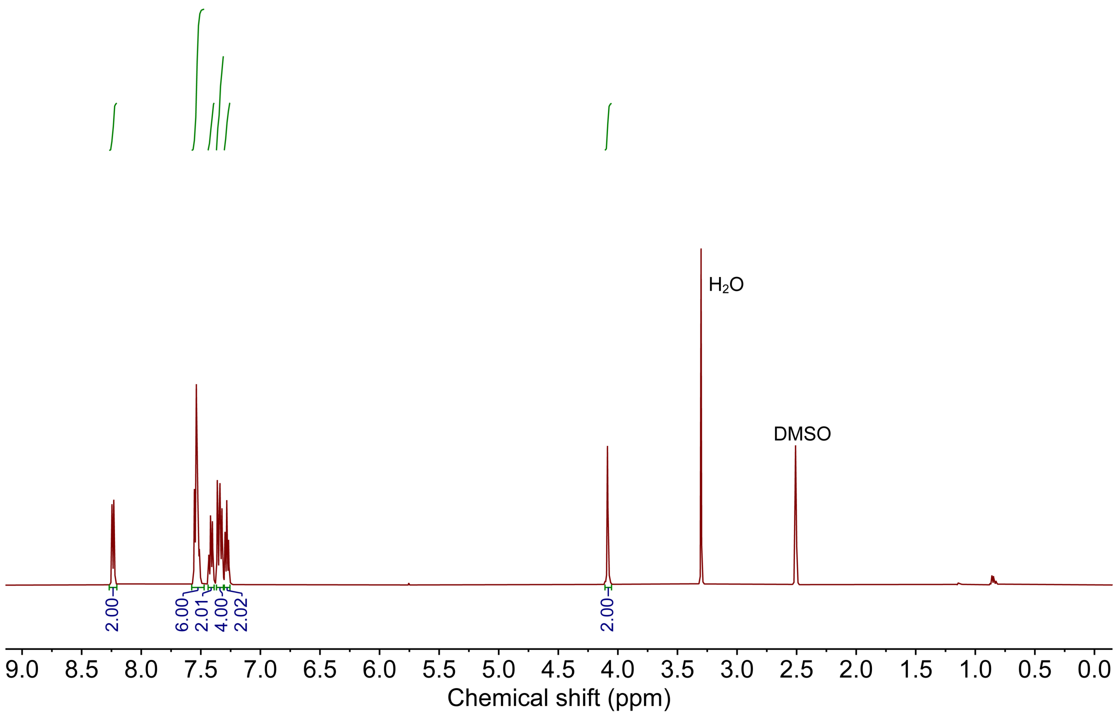

Figure S4. ^{1}H NMR spectrum of **Cz-C-PhBr** in *d*$_6$-DMSO.

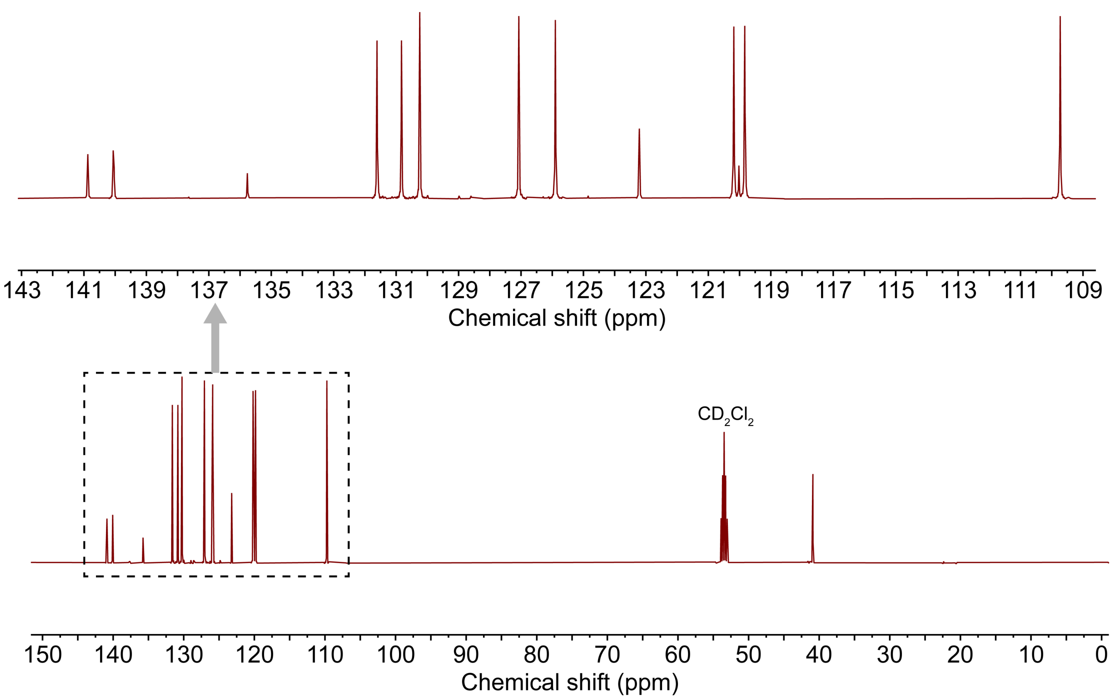

Figure S5. ^{13}C NMR spectrum of **Cz-C-PhBr** in CD_2Cl_2.

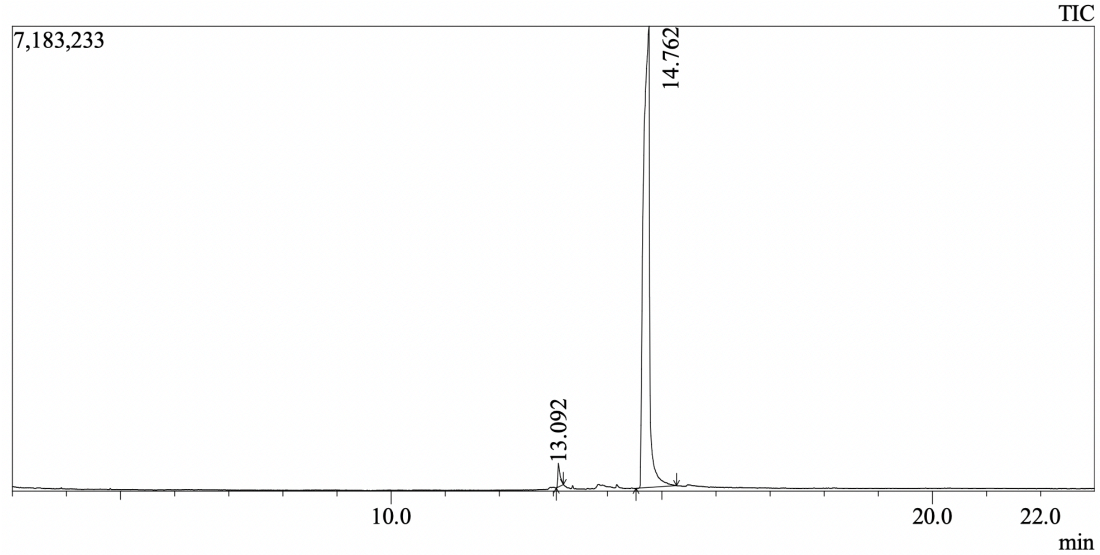

Peak Report TIC

Peak#	R.Time	Area	Area%	Height	A/H	Base m/z	Base Int.
1	13.092	1083120	1.86	362828	2.99	333.20	123703
2	14.762	57073522	98.14	7123158	8.01	411.10	1296240
		58156642	100.00	7485986			

Figure S6. GC-MS trace of **Cz-C-PhBr**.

Synthesis of 9-(4-(4-(4,4,5,5-tetramethyl-1,3,2-dioxaborolan-2-yl)benzyl)phenyl)-9H-carbazole (Cz-C-PhBpin)

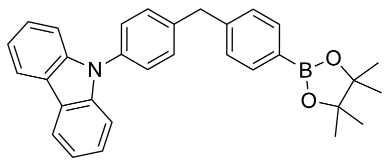

The synthetic route follows the Miyaura borylation reaction according to the literature.[25] **Cz-C-PhBr** (0.85 g, 2.06 mmol, 1.0 equiv.), bis(pinacolato)diboron (0.68 g, 2.68 mmol, 1.3 equiv.), potassium acetate (0.61 g, 6.18 mmol, 3.0 equiv.), 1,1'- Pd(dppf)Cl_2 (0.075 g, 0.10 mmol, 0.05 equiv.) were added to a Schlenk flask containing 10 mL of anhydrous 1,4-dioxane. After degassing the flask, the reaction system was placed under a nitrogen atmosphere. The reactant mixture was heated at 85 °C for 24 h. After cooling to room temperature, DCM (50 mL) was added to the mixture. The mixture was washed with a saturated NaCl aqueous solution (50 mL × 2). The collected organic phase was dried over anhydrous sodium sulfate and concentrated under reduced pressure. The collected crude product was purified by silica gel column chromatography (DCM: Hexane = 1:1, $\mathbf{R}_f$: 0.37) to afford the desired compound as a white solid. **Yield**: 0.32 g, 34%. **Mp**: 128-129 °C. **^{1}H NMR (400 MHz, *d_6*-DMSO) δ (ppm)**: 8.24 (d, J = 7.7 Hz, 2H), 7.70 – 7.64 (m, 2H), 7.57 – 7.48 (m, 4H), 7.44 – 7.33 (m, 6H), 7.28 (ddd, J = 7.9, 7.0, 1.2 Hz, 2H), 4.12 (s, 2H), 1.29 (s, 12H). **^{13}C NMR (126 MHz, CD_2Cl_2) δ (ppm)**: 144.14, 140.90, 140.51, 135.60, 135.00, 130.25, 128.46, 127.00, 125.88, 123.18, 120.15, 119.78, 109.76, 83.74, 41.74, 24.67. **GC-MS** (m/z): 459.30; retention time: 16.466 min.

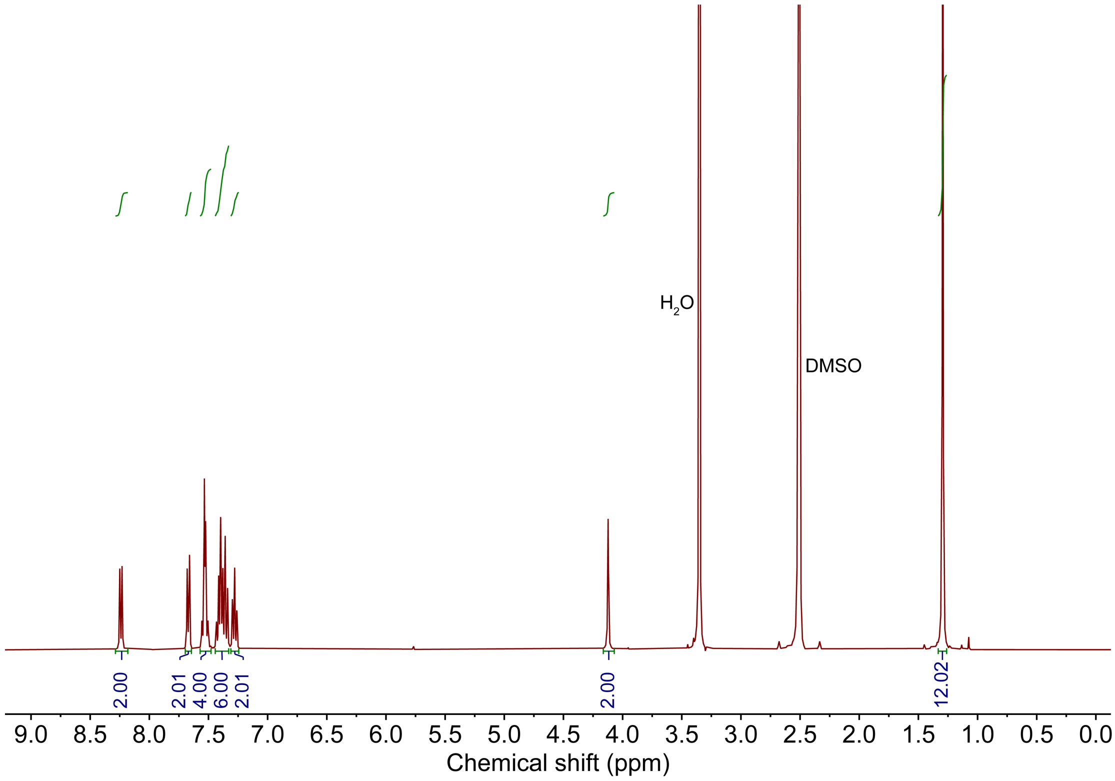

Figure S7. ^{1}H NMR spectrum of **Cz-C-PhBpin** in d_6-DMSO.

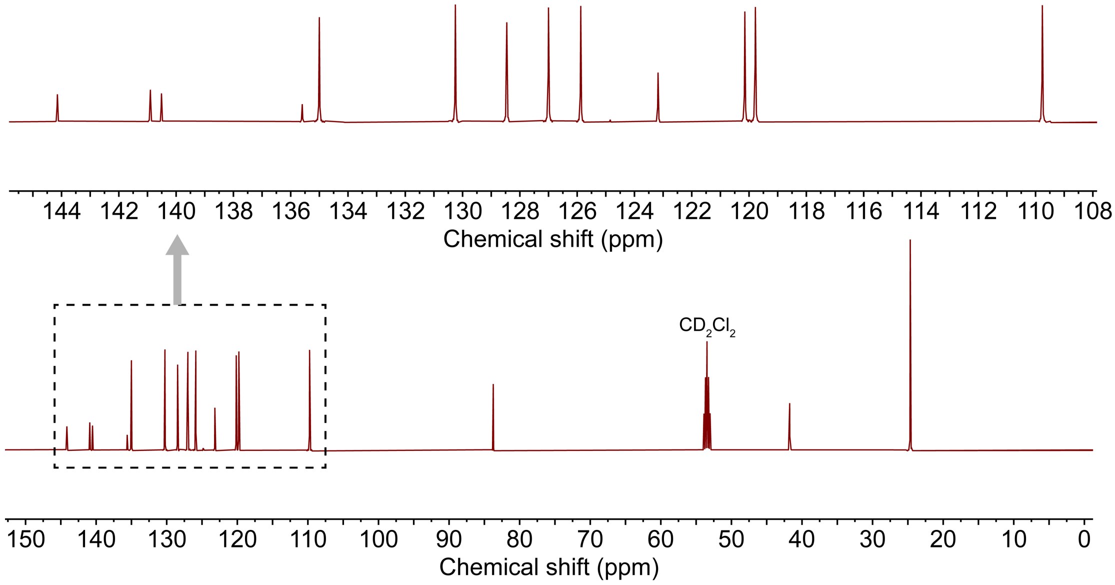

Figure S8. ^{13}C NMR spectrum of **Cz-C-PhBpin** in CD_2Cl_2.

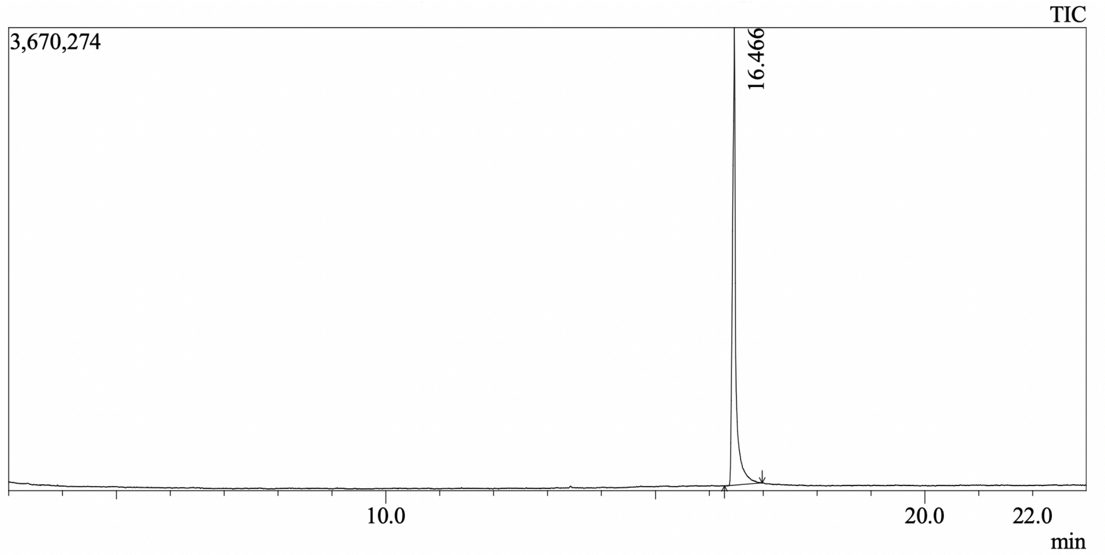

Peak Report TIC

Peak#	R.Time	Area	Area%	Height	A/H	Base m/z	Base Int.
1	16.466	15233628	100.00	3624982	4.20	459.30	1041345
		15233628	100.00	3624982			

Figure S9. GC-MS trace of **Cz-C-PhBpin**.

Synthesis of (4-(9H-carbazol-9-yl)phenyl)(4-(4,4,5,5-tetramethyl-1,3,2-dioxaborolan-2-yl)phenyl)methanone (Cz-CO-PhBpin)

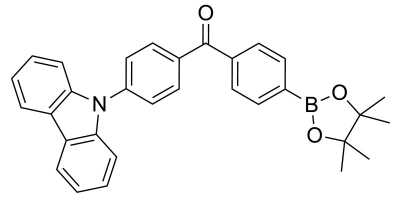

The synthetic route is similar to that of **Cz-C-PhBpin**, with **Cz-CO-PhBr** (0.85 g, 1.99 mmol, 1.0 equiv.) instead of **Cz-C-PhBr** as reactant. The collected crude product was purified by silica gel column chromatography (DCM: Hexane = 9:1, **R*f***: 0.32) to afford the desired compound as a white solid. **Yield**: 0.71 g, 75%. **Mp**: 173-174 °C. **^{1}H NMR (400 MHz, *d*$_6$-DMSO) δ (ppm)**: 8.32 – 8.25 (m, 2H), 8.09 – 8.02 (m, 2H), 7.94 – 7.83 (m, 6H), 7.57 (dt, J = 8.2, 0.9 Hz, 2H), 7.49 (ddd, J = 8.3, 7.1, 1.3 Hz, 2H), 7.35 (ddd, J = 7.9, 7.1, 1.0 Hz, 2H), 1.34 (s, 12H). **^{13}C NMR (126 MHz, CD_2Cl_2) δ (ppm)**: 195.47, 141.59, 140.29, 139.63, 135.90, 134.55, 131.84, 128.95, 126.26, 126.20, 123.74, 120.53, 120.35, 109.84, 84.26, 24.70. **GC-MS** (m/z): 473.30; retention time: 18.113 min.

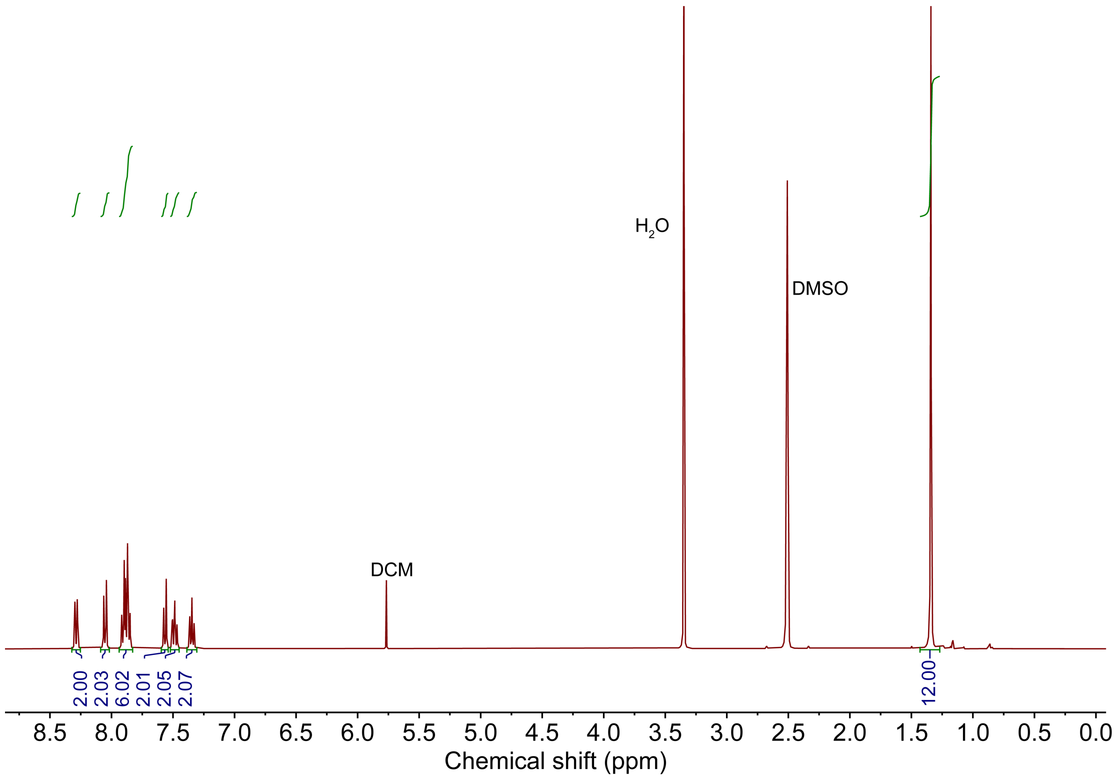

Figure S10. ^{1}H NMR spectrum of **Cz-CO-PhBpin** in d_6-DMSO.

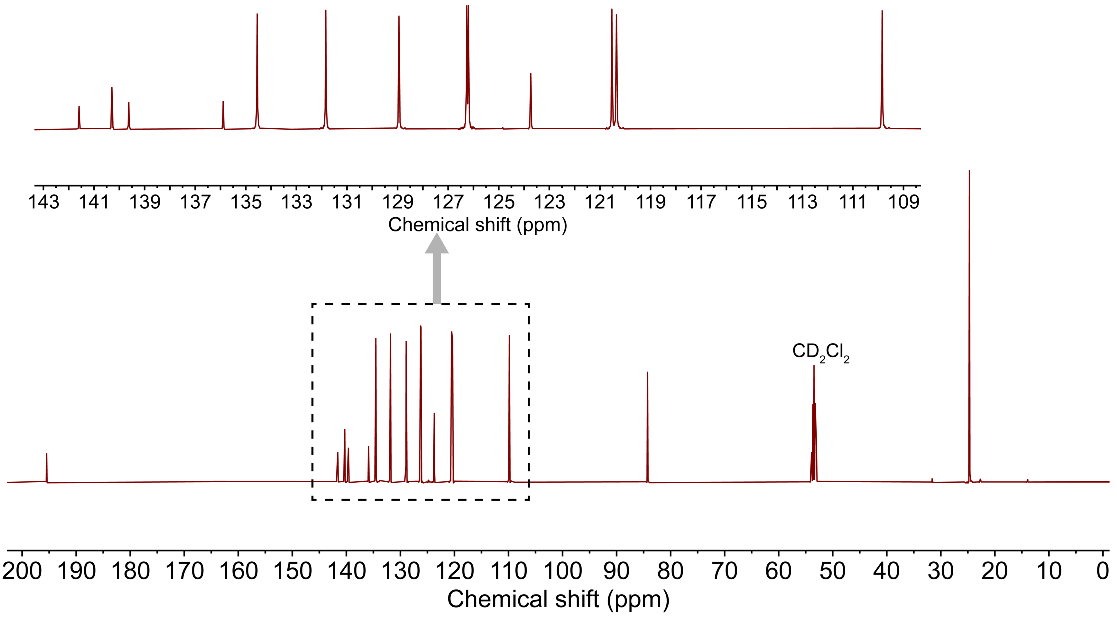

Figure S11. ^{13}C NMR spectrum of **Cz-CO-PhBpin** in CD_2Cl_2.

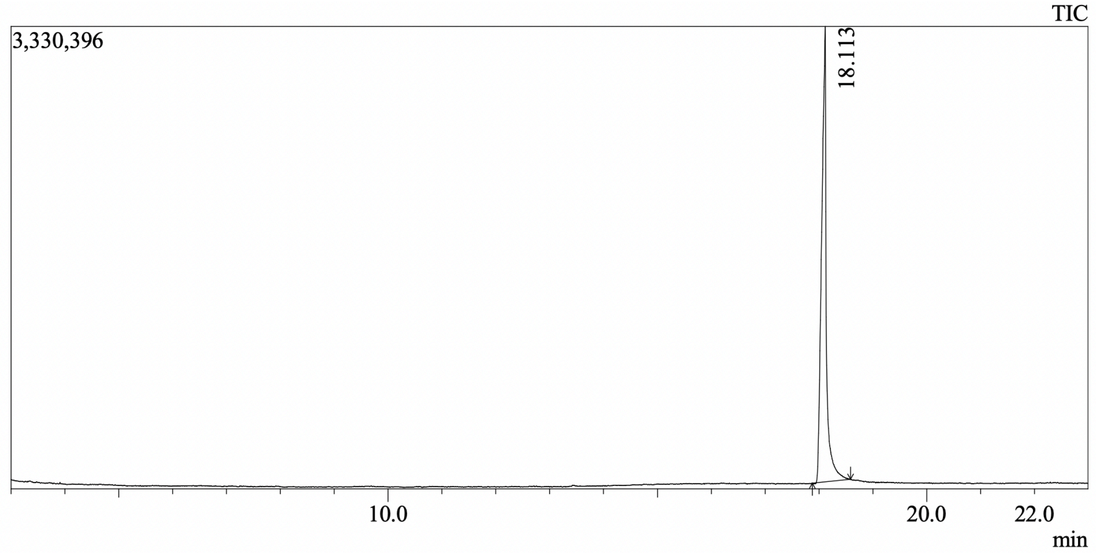

Peak Report TIC

Peak#	R.Time	Area	Area%	Height	A/H	Base m/z	Base Int.
1	18.113	20165403	100.00	3279370	6.15	473.30	878782
		20165403	100.00	3279370			

Figure S12. GC-MS trace of **Cz-CO-PhBpin**.

Synthesis of 10-(4-(4-(9H-carbazol-9-yl)benzyl)phenyl)-10*H*-phenothiazine 5,5-dioxide ($Cz-C-PTZSO_2$)

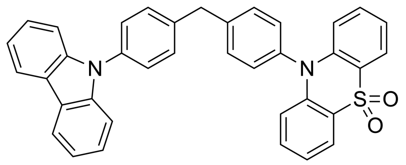

The synthetic route follows the Ullmann coupling reaction according to the literature.[24] **Cz-C-PhBr** (0.80 g, 1.94 mmol, 1.0 equiv.), 10*H*-phenothiazine 5,5-dioxide (0.54 g, 2.33 mmol, 1.2 equiv.), potassium carbonate (0.40 g, 2.91 mmol, 1.5 equiv.), copper iodide (0.037 g, 0.19 mmol, 0.1 equiv.), and 2,2,6,6-tetramethylheptane-3,5-dione (0.072 g, 0.39 mmol, 0.2 equiv.) were added to a Schlenk flask containing 12 mL of anhydrous DMF. After degassing the flask, the reaction system was placed under a nitrogen atmosphere. The reactant mixture was heated at 150 °C for 24 h. After cooling to room temperature, DCM (50 mL) was added to the mixture. The organic solution was then washed with a saturated NaCl aqueous solution (50 mL × 2) and further with a 1 M HCl solution (50 mL). The collected organic phase was dried over anhydrous magnesium sulfate and concentrated under reduced pressure. The collected crude product was purified by silica gel column chromatography (DCM, $\mathbf{R}_f$: 0.32) and further by recrystallization in toluene to afford the desired compound as a white crystal. **Yield**: 0.99 g, 91%. **Mp**: 244-245 °C. **^{1}H NMR (500 MHz, d_6-DMSO) δ (ppm)**: 8.26 (dt, J = 7.8, 1.0 Hz, 2H), 8.09 (dd, J = 8.0, 1.6 Hz, 2H), 7.78 – 7.71 (m, 2H), 7.68 (d, J = 8.4 Hz, 2H),

7.65 – 7.60 (m, 2H), 7.57 (ddd, J = 8.8, 7.2, 1.6 Hz, 2H), 7.51 – 7.46 (m, 2H), 7.46 – 7.38 (m, 4H), 7.35 (ddd, J = 8.0, 7.2, 0.9 Hz, 2H), 7.30 (ddd, J = 7.9, 6.7, 1.4 Hz, 2H), 6.65 (d, J = 8.6 Hz, 2H), 4.31 (s, 2H). **^{13}C NMR (126 MHz, *d_6*-DMSO) δ (ppm)**: 143.25, 140.68, 140.59, 140.44, 136.84, 135.54, 133.95, 132.36, 131.13, 130.79, 127.32, 126.71, 123.28, 123.15, 122.76, 122.45, 121.01, 120.49, 117.75, 110.15, 40.69. **HR-MS [M+H]$^+$ Calculated**: ($C_{37}H_{26}N_2O_2S$) 563.1788; **Found**: 563.1763. **Anal. Calcd. for $C_{37}H_{26}N_2O_2S$**: C, 78.98%; H, 4.66%; N, 4.98%. **Found**: C, 78.30%; H, 4.59%; N, 5.02% (Note: despite 6 tries this is the best EA found). **HPLC purity**: > 99.99% (retention time: 8.09 minutes in 75% MeCN/25% H_2O).

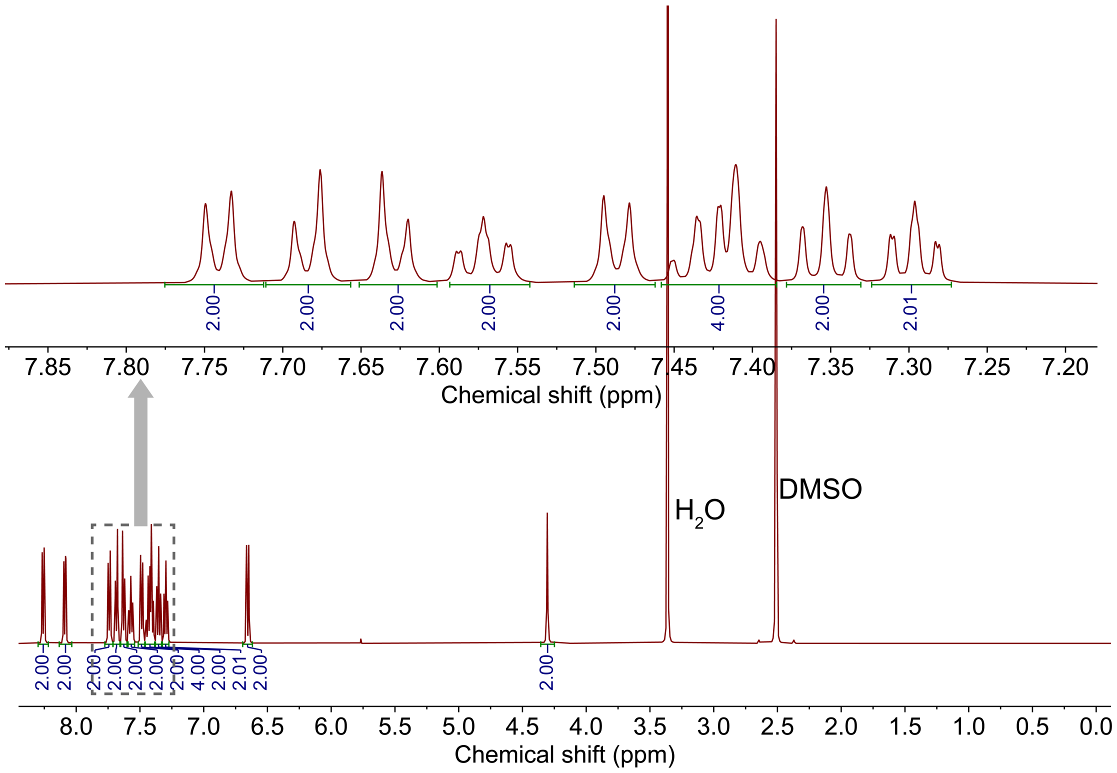

Figure S13. ^{1}H NMR spectrum of **Cz-C-PTZSO$_2$** in *d_6*-DMSO.

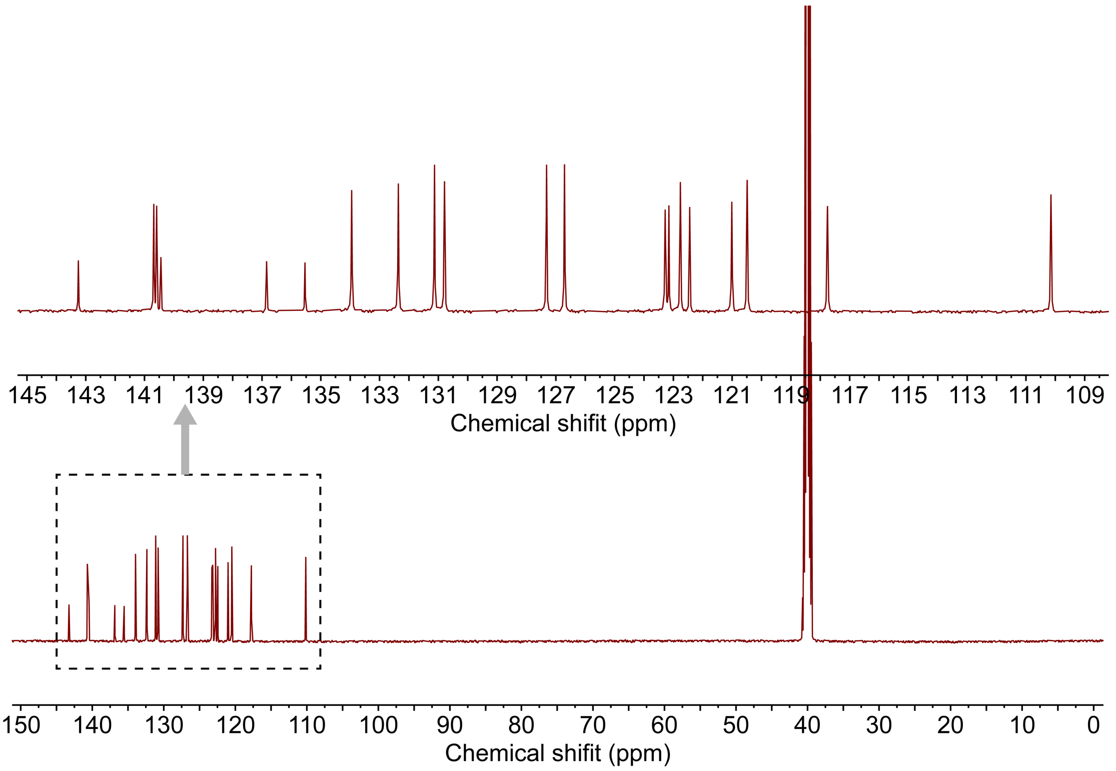

Figure S14. ^{13}C NMR spectrum of **Cz-C-PTZSO$_2$** in d_6-DMSO.

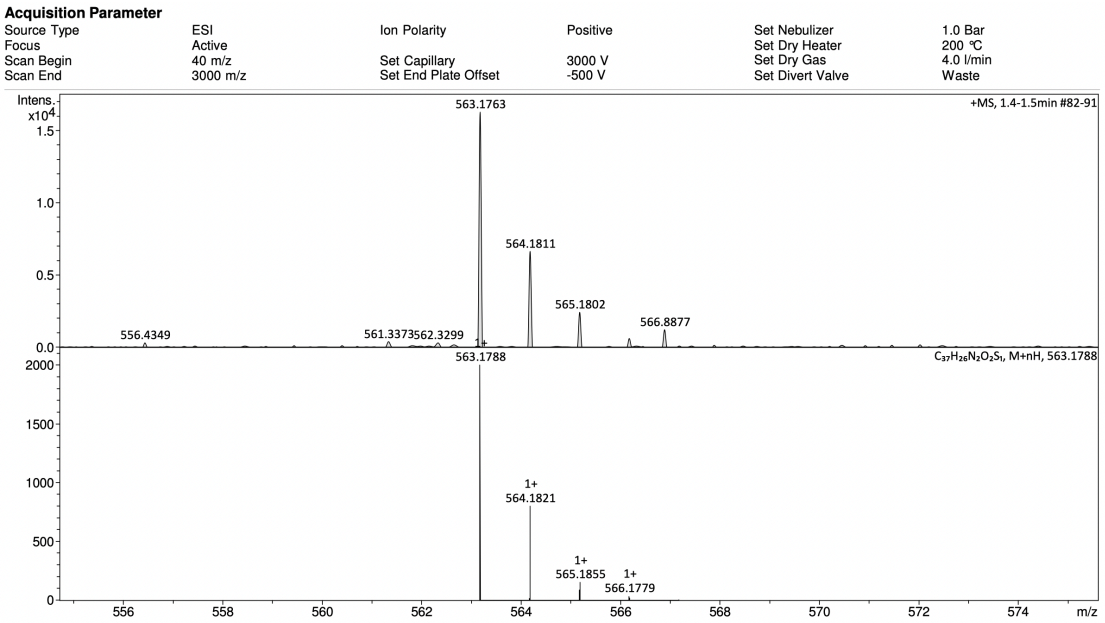

Figure S15. HRMS (ESI) of **Cz-C-PTZSO$_2$**.

Elemental Analysis Service Request Form

Researcher name Tao Wang

Researcher email tw72@st-andrews.ac.uk

NOTE: Please submit ca. 10 mg of sample

**Sample reference number**	WT-2022-3
**Name of Compound**	
**Molecular formula**	C37H26N2O2S
**Stability**	Stable in air and in most solvents
**Hazards**	
**Other Remarks**	

Analysis type:

Single ☐ **Duplicate** ■ **Triplicate** ☐

Analysis Result:

Element	Expected %	Found (1)	Found (2)	Found (3)
Nitrogen	4.98	5.02	5.13	
Carbon	78.98	78.30	78.26	
Hydrogen	4.66	4.59	4.60	

Authorising Signature:

**Date completed**	25·08·22
**Signature**	[illegible]
**comments**	

Figure S16. Elemental analysis result of **Cz-C-PTZSO$_2$**.

HPLC Trace Report10Apr2022

<Sample Information>

Sample Name	: CZ-C-PTZSO2		
Sample ID	:		
Method Filename	: 75% Acetonitrile 25 Water 20 mins.lcm		
Batch Filename	: Cz-linker-Acceptor.lcb		
Vial #	: 1-2	Sample Type	: Unknown
Injection Volume	: 10 uL		
Date Acquired	: 10/04/2022 19:10:56	Acquired by	: System Administrator
Date Processed	: 10/04/2022 19:30:57	Processed by	: System Administrator

<Chromatogram>

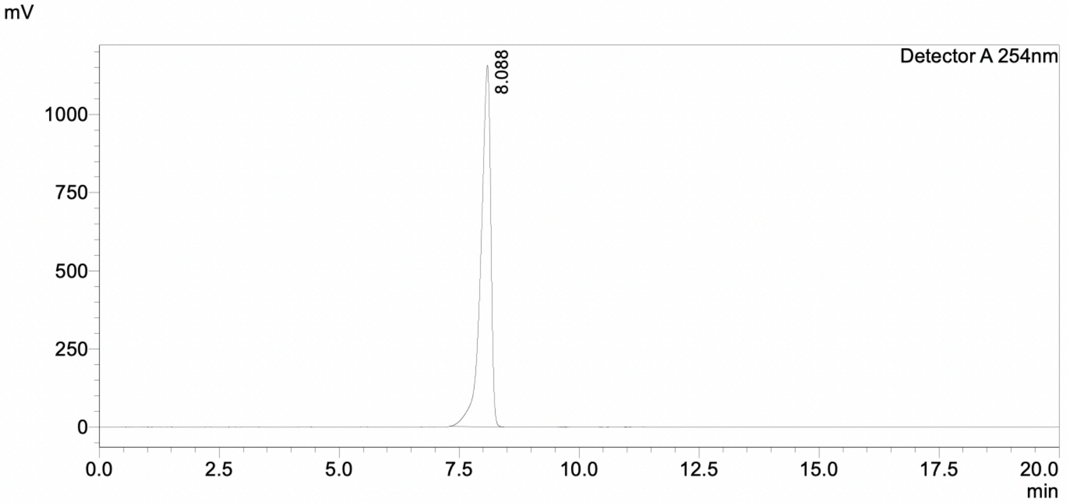

<Peak Table>

Detector A 254nm

Peak#	Ret. Time	Area	Height	Area%	Area/Height	Width at 5% Height
1	8.088	16665703	1155442	100.000	14.424	0.555
Total		16665703	1155442	100.000		

Figure S17. HPLC trace of **Cz-C-PTZSO$_2$**.

Synthesis of (4-(9H-carbazol-9-yl)phenyl)(4-(5,5-dioxido-10*H*-phenothiazin-10-yl)phenyl)methanone (Cz-CO-PTZSO$_2$)

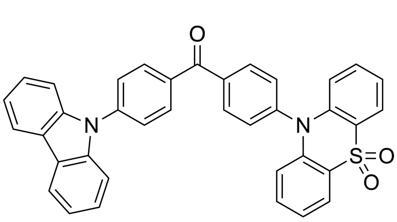

The synthetic route is similar to that of **Cz-C-PTZSO$_2$** with **Cz-CO-PhBr** (0.90 g, 2.11 mmol, 1.0 equiv.) instead of **Cz-C-PhBr**. The collected crude product was purified by silica gel column chromatography (DCM, **R$_f$**: 0.18) and further by sublimation to afford the desired compound as a white solid. **Yield**: 1.07 g, 88%. **Mp**: 270-272 °C. **^{1}H NMR (500 MHz, *d$_6$*-DMSO) δ (ppm)**: 8.30 (dt, J = 7.7, 1.0 Hz, 2H), 8.26 – 8.20 (m, 4H), 8.13 (dd, J = 7.9, 1.6 Hz, 2H), 7.96 – 7.92 (m, 2H), 7.80 – 7.76 (m, 2H), 7.65 – 7.58 (m,

4H), 7.50 (ddd, J = 8.3, 7.1, 1.2 Hz, 2H), 7.38 (dddd, J = 22.9, 8.0, 7.2, 0.9 Hz, 4H), 6.77 (dd, J = 8.8, 0.9 Hz, 2H). **^{13}C NMR (126 MHz, *d_6*-DMSO) δ (ppm)**: 194.59, 142.34, 141.62, 140.33, 140.02, 138.68, 135.38, 134.07, 133.29, 132.51, 131.29, 127.00, 126.80, 123.69, 123.38, 123.04, 122.64, 121.21, 121.17, 117.87, 110.38. **HR-MS [M+H]$^+$ Calculated**: ($C_{37}H_{24}N_2O_3S$) 577.1576; **Found**: 577.1580. **Anal. Calcd. for $C_{37}H_{24}N_2O_3S$**: C, 77.06%; H, 4.20%; N, 4.86%. **Found**: C, 77.45; H, 4.34%; N, 4.92%. **HPLC purity**: 99.98% (retention time: 5.52 minutes in 75% MeCN/25% H_2O).

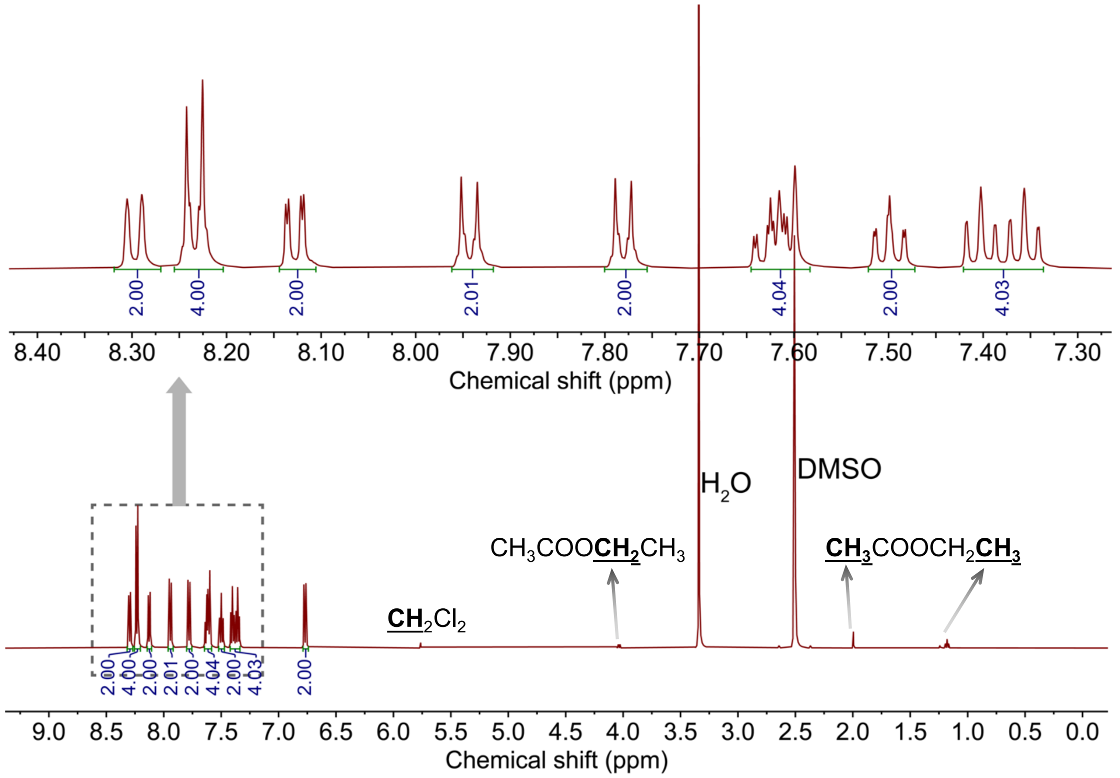

Figure S18. ^{1}H NMR spectrum of **Cz-CO-PTZSO$_2$** in *d_6*-DMSO.

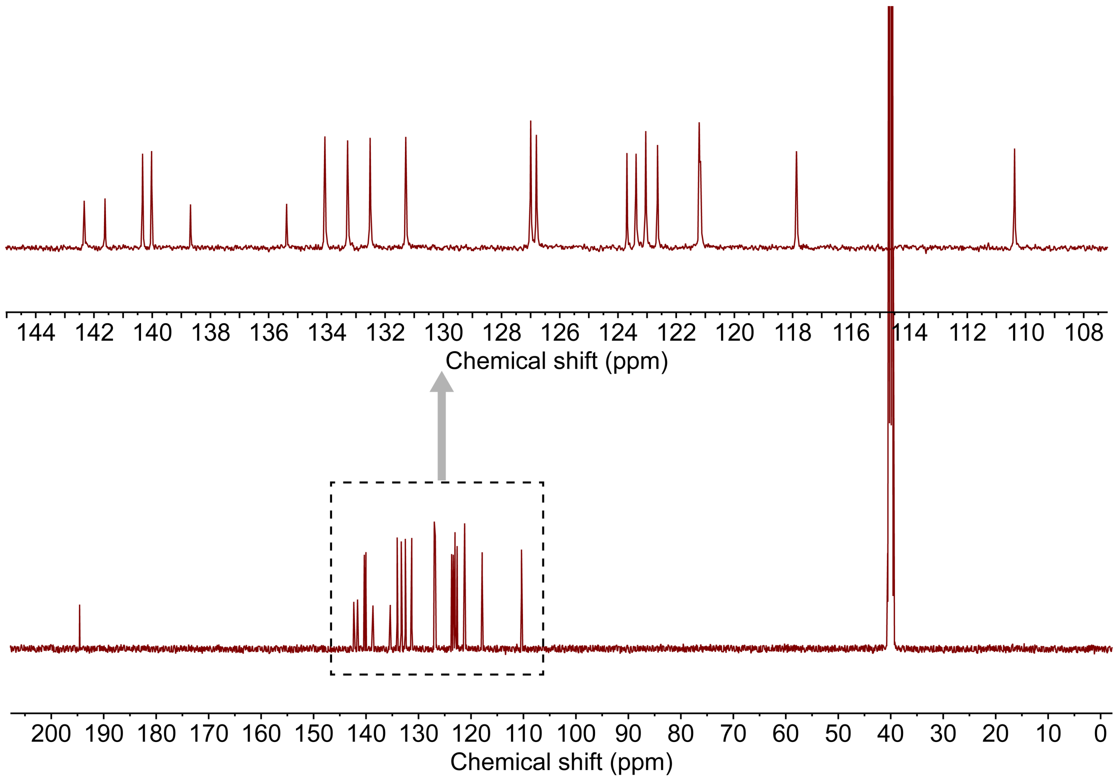

Figure S19. ^{13}C NMR spectrum of **Cz-CO-PTZSO$_2$** in d_6-DMSO.

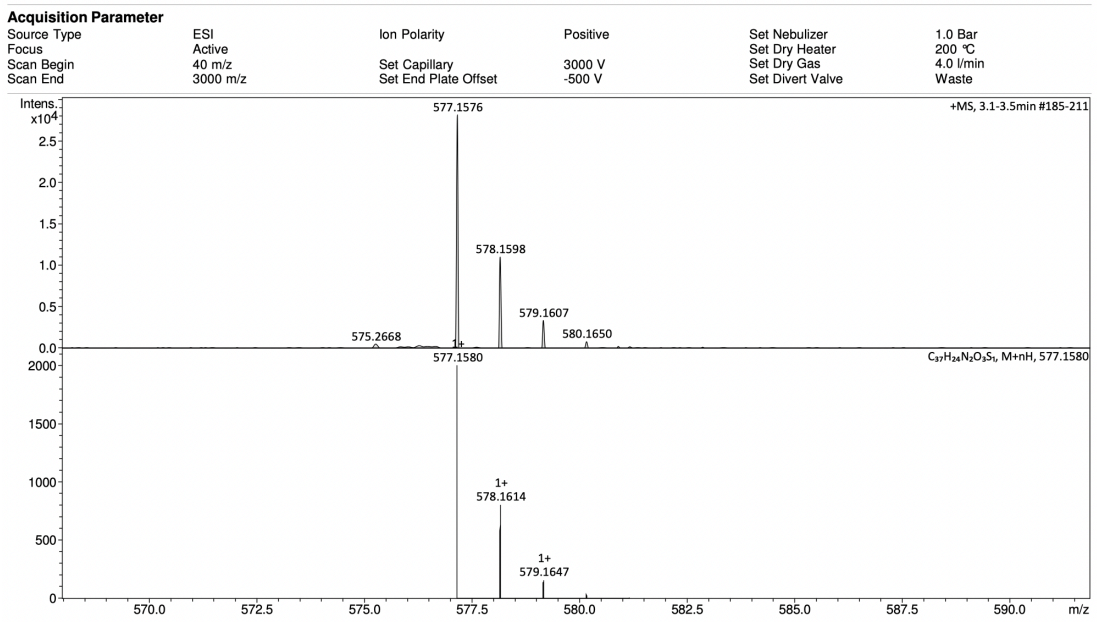

Figure S20. HRMS (ESI) of **Cz-CO-PTZSO$_2$**.

Elemental Analysis Service Request Form

Researcher name Tao Wang

Researcher email tw72@st-andrews.ac.uk

NOTE: Please submit ca. 10 mg of sample

**Sample reference number**	Cz-CO-PTZSO2
**Name of Compound**	
**Molecular formula**	C37H26N2O3S
**Stability**	Stable in air and in most solvents
**Hazards**	
**Other Remarks**	

Analysis type:

Single ☐ **Duplicate** ☑ **Triplicate** ☐

Analysis Result:

Element	Expected %	Found (1)	Found (2)	Found (3)
Carbon	77.06	77.60	77.45	
Hydrogen	4.20	4.36	4.34	
Nitrogen	4.86	4.88	4.92	
Oxygen				

Authorising Signature:

**Date completed**	29.04.22
**Signature**	[illegible]

Figure S21. Elemental analysis result of **Cz-CO-PTZSO$_2$**.

HPLC Trace Report06Jul2022

<Sample Information>

Sample Name : CZ-CO-PTZSO2
Sample ID :
Method Filename : 75% Acetonitrile 25 Water 20 mins.lcm
Batch Filename : Cz-linker-Acceptor.lcb
Vial # : 1-3 Sample Type : Unknown
Injection Volume : 5 uL
Date Acquired : 10/04/2022 20:15:24 Acquired by : System Administrator
Date Processed : 10/04/2022 20:35:26 Processed by : System Administrator

<Chromatogram>

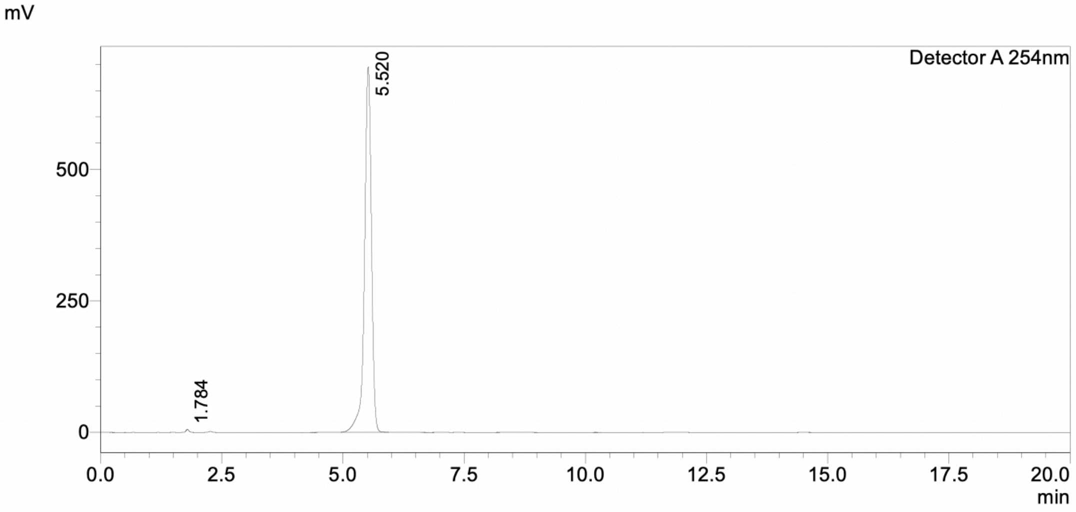

<Peak Table>

Detector A 254nm

Peak#	Ret. Time	Area	Height	Area%	Area/Height	Width at 5% Height
1	1.784	1578	544	0.023	2.901	--
2	5.520	6914657	694462	99.977	9.957	0.372
Total		6916235	695006	100.000		

Figure S22. HPLC trace of **Cz-CO-PTZSO$_2$**.

Synthesis of 9-(4-(4-(4,6-diphenyl-1,3,5-triazin-2-yl)benzyl)phenyl)-9H-carbazole (Cz-C-TRZ)

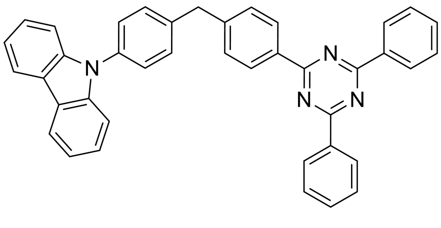

The synthetic route follows the Suzuki-Miyaura coupling reaction according to the literature.[26] **Cz-C-PhBpin** (0.30 g, 0.65 mmol, 1.0 equiv.), 2-chloro-4,6-diphenyl-1,3,5-triazine (0.23 g, 0.85 mmol, 1.3 equiv.), Pd(PPh$_3$)$_4$ (0.034 g, 0.033 mmol, 0.05 equiv.) were added to a Schlenk flask containing 10 mL of degassed THF. Then the degassed potassium carbonate water solution (1 mL, 2 mol/L) was added to the flask. The reactant mixture was heated at 75 °C for 24 h under the protection of N$_2$. After cooling to

room temperature, DCM (50 mL) was added to the mixture. The organic solution was then washed with a saturated NaCl aqueous solution (50 mL × 2) and further with a 1 M HCl solution (50 mL). The collected organic phase was dried over anhydrous magnesium sulfate and concentrated under reduced pressure. The collected crude product was purified by silica gel column chromatography (DCM: Hexane = 1:1, **R*f***: 0.50) and further by recrystallization in toluene to afford the desired compound as a white crystalline solid. **Yield**: 0.32 g, 86%. **Mp**: 253-254 °C. **^{1}H NMR (500 MHz, $CDCl_3$) δ (ppm)**: 8.85 – 8.75 (m, 6H), 8.17 (dt, J = 7.8, 1.0 Hz, 2H), 7.67 – 7.59 (m, 6H), 7.56 – 7.51 (m, 4H), 7.51 – 7.47 (m, 2H), 7.46 – 7.40 (m, 4H), 7.31 (ddd, J = 8.0, 5.4, 2.7 Hz, 2H), 4.27 (s, 2H). **^{13}C NMR (101 MHz, CD_2Cl_2) δ (ppm)**: 171.60, 171.53, 145.94, 140.90, 140.08, 136.27, 135.79, 134.45, 132.56, 130.38, 129.42, 129.25, 128.86, 128.67, 127.11, 125.89, 123.20, 120.16, 119.81, 109.75, 41.61. **HR-MS $[M+H]^+$ Calculated**: ($C_{40}H_{28}N_4$) 565.2356; **Found**: 565.2387. **Anal. Calcd. for $C_{40}H_{28}N_4$**: C,85.08%; H, 5.00%; N, 9.92%. **Found**: C, 85.27; H, 5.06%; N, 9.39%. **HPLC purity**: > 99.99% (retention time: 10.93 minutes in THF).

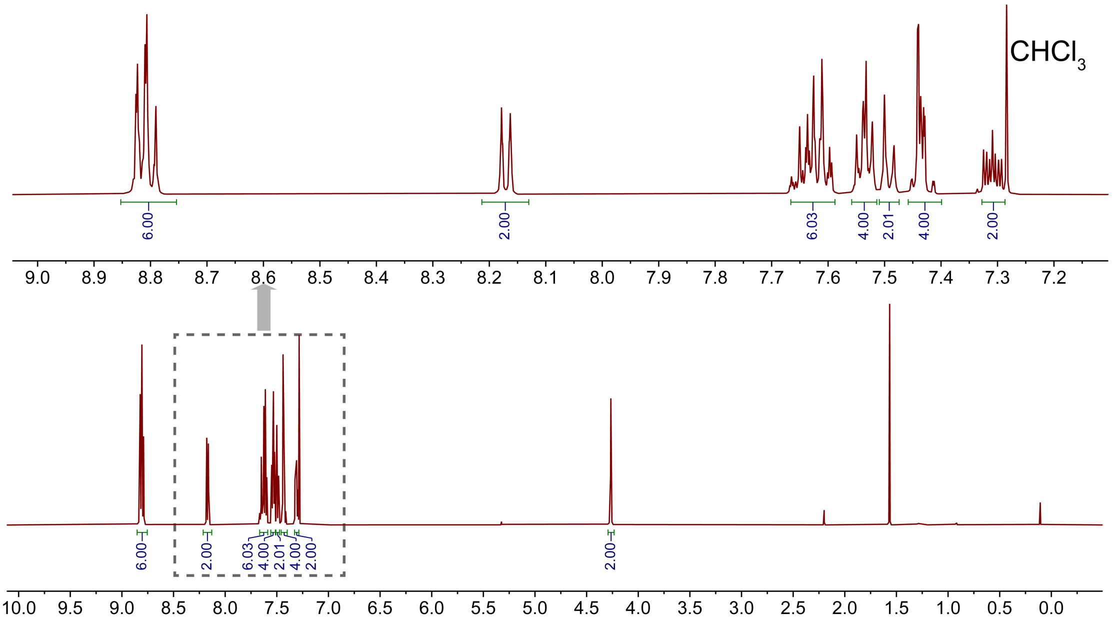

Figure S23. ^{1}H NMR spectrum of **Cz-C-TRZ** in $CDCl_3$.

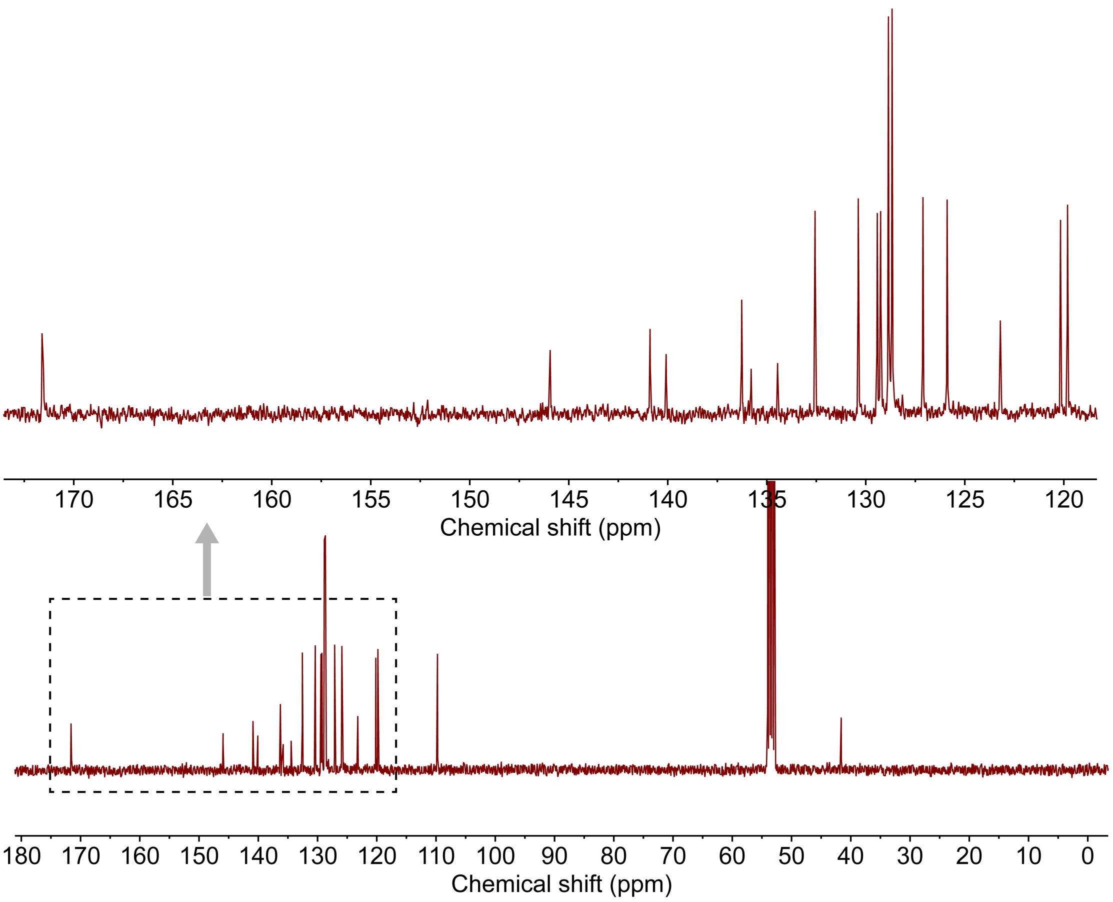

Figure S24. ^{13}C NMR spectrum of **Cz-C-TRZ** in CD_2Cl_2.

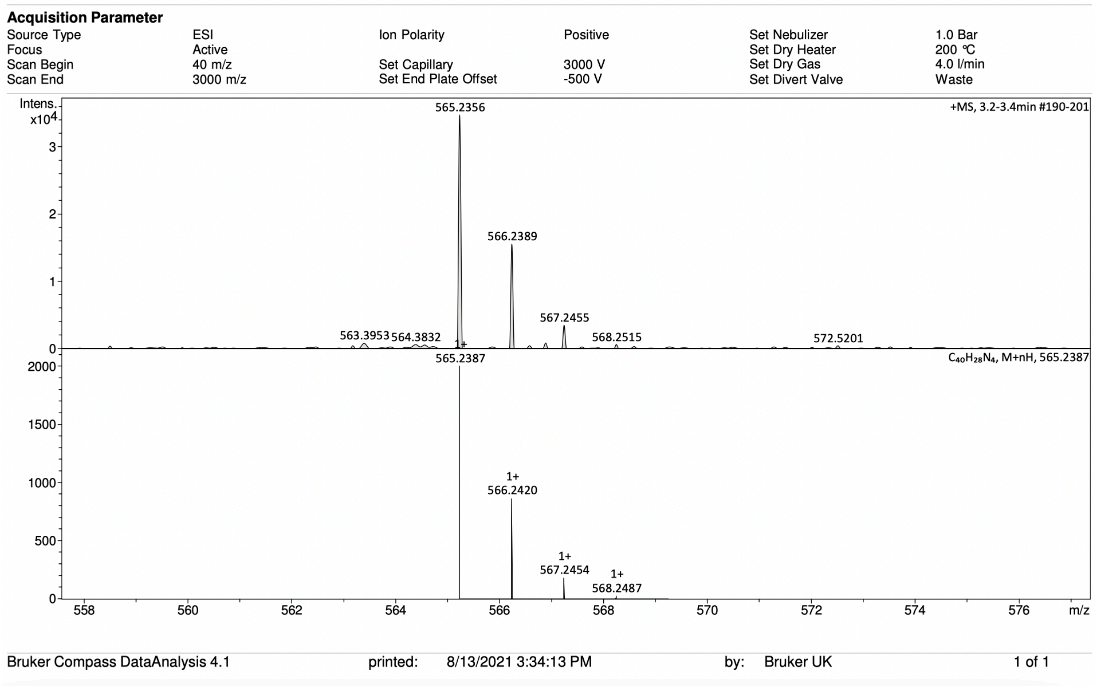

Figure S25. HRMS (ESI) of **Cz-C-TRZ**.

Elemental Analysis Service Request Form

Researcher name Tao Wang

Researcher email tw72@st-andrews.ac.uk

NOTE: Please submit ca. 10 mg of sample

**Sample reference number**	TW-I108-2nd
**Name of Compound**	
**Molecular formula**	C40H28N4
**Stability**	Stable in air and in most solvents
**Hazards**	
**Other Remarks**	

Analysis type:

Single ☐ **Duplicate** ☑ **Triplicate** ☐

Analysis Result:

Element	Expected %	Found (1)	Found (2)	Found (3)
Carbon	85.08	85.27	85.99	
Hydrogen	5.00	5.06	5.09	
Nitrogen	9.92	9.39	9.48	
Oxygen				

Authorising Signature:

**Date completed**	15.12.21
**Signature**	[illegible]

Figure S26. Elemental analysis result of **Cz-C-TRZ**

HPLC Trace Report15Jun2022

<Sample Information>

Sample Name	: Cz-C-TRZ		
Sample ID	:		
Method Filename	: 100% THF 20 mins 280nm - new-please use.lcm		
Batch Filename	: Cz-C CO-TRZ THF.lcb		
Vial #	: 1-2	Sample Type	: Unknown
Injection Volume	: 10 uL		
Date Acquired	: 15/06/2022 19:28:06	Acquired by	: System Administrator
Date Processed	: 15/06/2022 19:48:08	Processed by	: System Administrator

<Chromatogram>

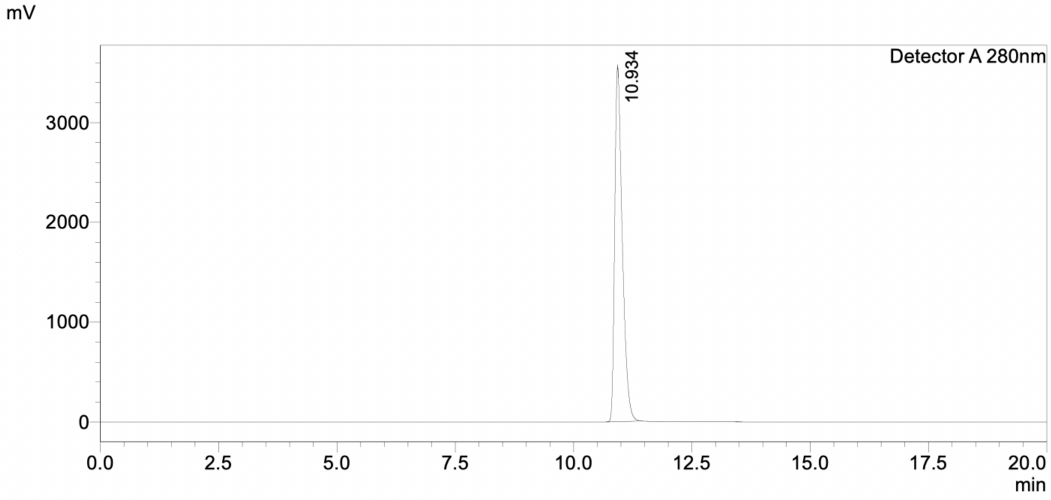

<Peak Table>

Detector A 280nm

Peak#	Ret. Time	Area	Height	Area%	Area/Height	Width at 5% Height
1	10.934	39629769	3556685	100.000	11.142	0.388
Total		39629769	3556685	100.000		

Figure S27. HPLC trace of **Cz-C-TRZ**

Synthesis of (4-(9H-carbazol-9-yl)phenyl)(4-(4,6-diphenyl-1,3,5-triazin-2-yl)phenyl)methanone (Cz-CO-TRZ)

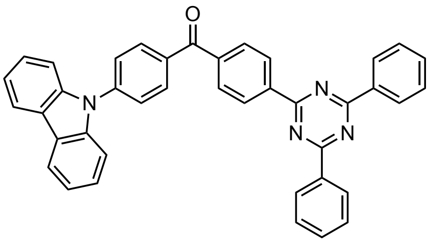

The synthetic route is similar to that of **Cz-C-TRZ**, with **Cz-CO-PhBpin** (0.60 g, 1.27 mmol, 1.0 equiv.) instead of **Cz-C-PhBpin**. The collected crude product was purified by silica gel column chromatography (DCM: Hexane = 1:1, **R*f***: 0.38) and further by sublimation to afford the desired compound as a yellow solid. **Yield**: 0.66 g, 90%. **Mp**: 258-259 °C. **^{1}H NMR (500 MHz, $CDCl_3$) δ (ppm)**: 8.98 (d, J = 8.1 Hz, 2H), 8.87 – 8.80 (m, 4H), 8.19 (t, J = 8.5 Hz, 4H), 8.14 – 8.10

(m, 2H), 7.84 – 7.79 (m, 2H), 7.70 – 7.61 (m, 6H), 7.59 (d, J = 8.2 Hz, 2H), 7.49 (tt, J = 8.3, 1.3 Hz, 2H), 7.37 (t, J = 7.4 Hz, 2H). **^{13}C NMR (101 MHz, CDCl$_3$) δ (ppm)**: 195.23, 171.91, 170.77, 142.03, 140.58, 140.22, 139.94, 135.96, 135.65, 132.81, 132.03, 130.19, 129.07, 128.99, 128.76, 126.36, 126.29, 123.91, 120.71, 120.54, 109.83. **HR-MS [M+H]$^+$ Calculated**: ($C_{40}H_{26}N_4O$) 579.2179; **Found**: 579.2165. **Anal. Calcd. for $C_{40}H_{26}N_4O$**: C, 83.02%; H, 4.53%; N, 9.68%. **Found**: C, 83.07; H, 4.66%; N, 9.73%. **HPLC purity**: > 99.99% (retention time: 10.91 minutes in THF).

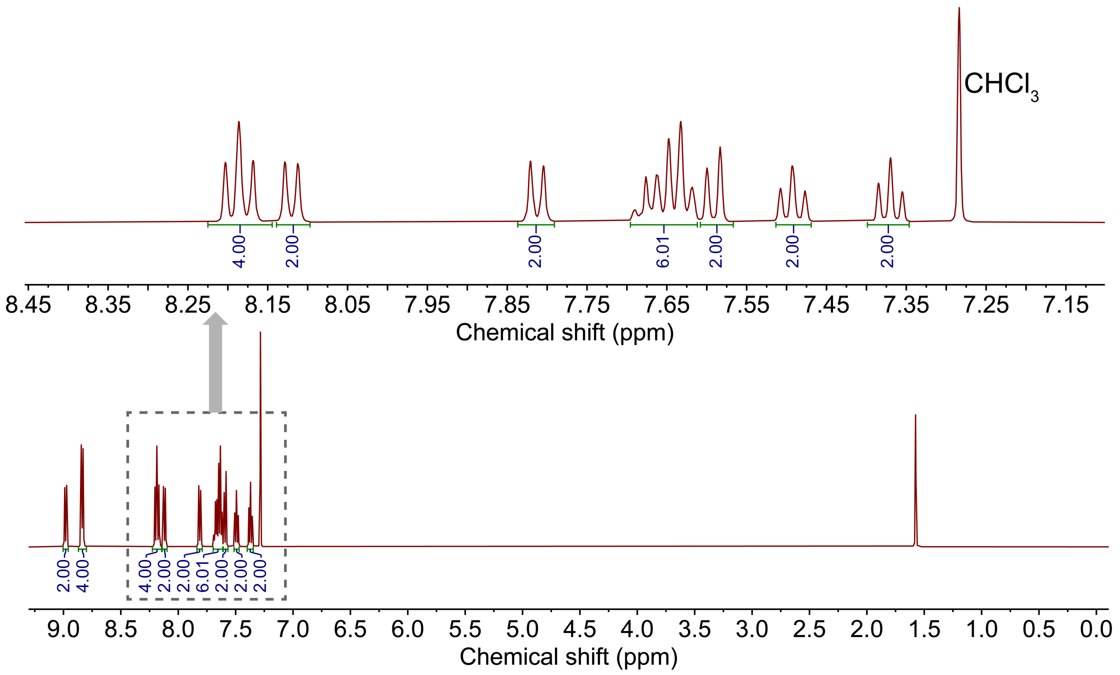

Figure S28. ^{1}H NMR spectrum of **Cz-CO-TRZ** in $CDCl_3$

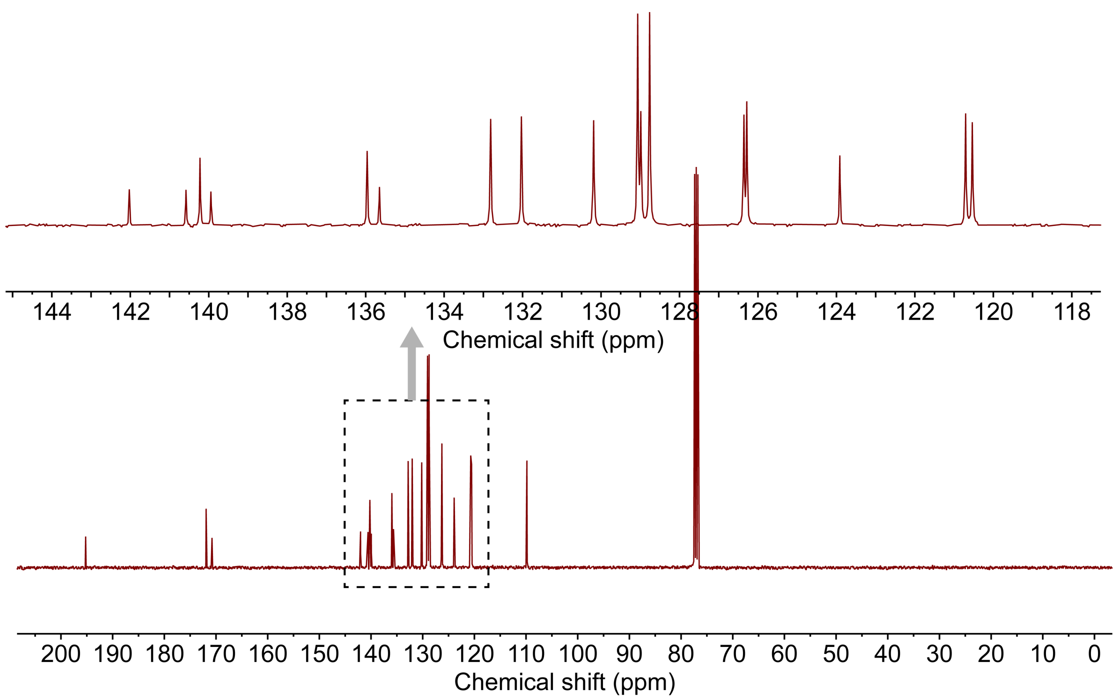

Figure S29. ^{13}C NMR spectrum of **Cz-CO-TRZ** in $CDCl_3$.

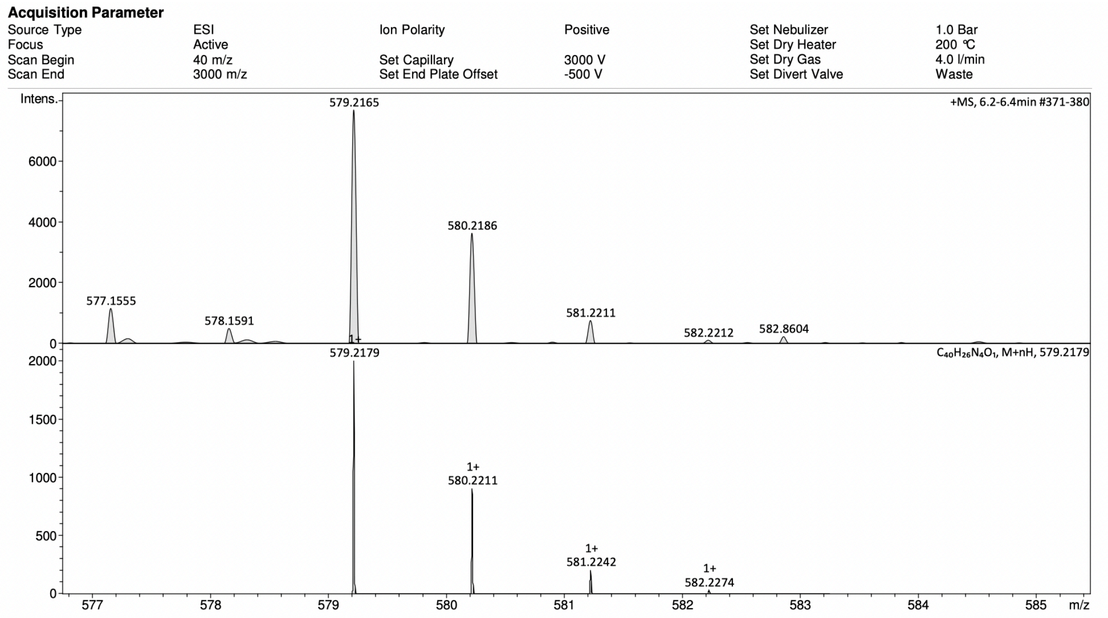

Figure S30. HRMS (ESI) of **Cz-CO-TRZ**.

Elemental Analysis Service Request Form

Researcher name Tao Wang

Researcher email tw72@st-andrews.ac.uk

NOTE: Please submit ca. 10 mg of sample

**Sample reference number**	TW-I103
**Name of Compound**	
**Molecular formula**	C40H26N4O
**Stability**	Stable in air and in most solvents
**Hazards**	
**Other Remarks**	

Analysis type:

Single ☐ **Duplicate** ☒ **Triplicate** ☐

Analysis Result:

Element	Expected %	Found (1)	Found (2)	Found (3)
Carbon	83.02	83.68	83.07	
Hydrogen	4.53	4.63	4.66	
Nitrogen	9.68	9.81	9.73	
Oxygen				

Authorising Signature:

**Date completed**	05.11.21
**Signature**	J-P L

Figure S31. Elemental analysis result of **Cz-CO-TRZ**.

HPLC Trace Report15Jun2022

<Sample Information>

Sample Name	: Cz-CO-TRZ		
Sample ID	:		
Method Filename	: 100% THF 20 mins 280nm - new-please use.lcm		
Batch Filename	: Cz-C CO-TRZ THF.lcb		
Vial #	: 1-3	Sample Type	: Unknown
Injection Volume	: 10 uL		
Date Acquired	: 15/06/2022 20:08:55	Acquired by	: System Administrator
Date Processed	: 15/06/2022 20:28:56	Processed by	: System Administrator

<Chromatogram>

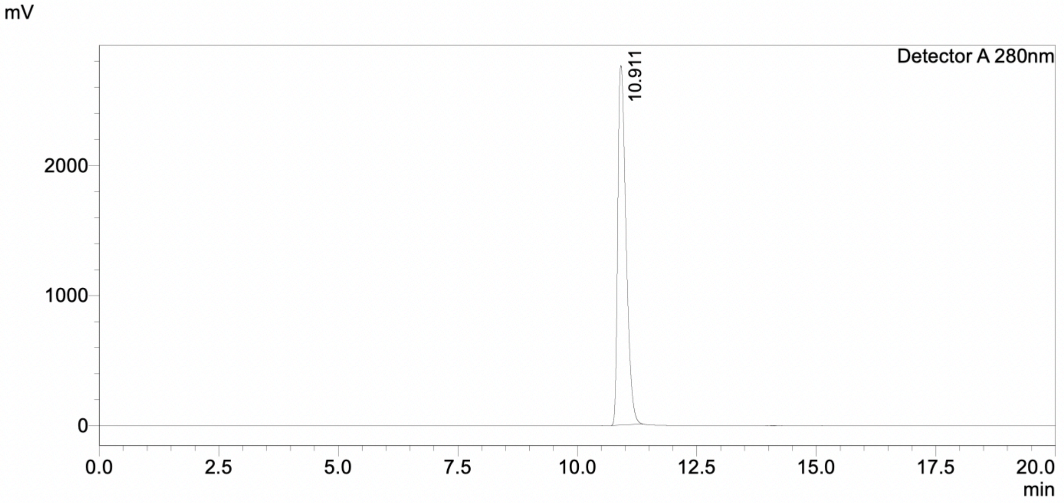

<Peak Table>

Detector A 280nm

Peak#	Ret. Time	Area	Height	Area%	Area/Height	Width at 5% Height
1	10.911	33266269	2762199	100.000	12.043	0.399
Total		33266269	2762199	100.000		

Figure S32. HPLC trace of **Cz-CO-TRZ**.

Photophysical data

Table S1. Electrochemical data.

	E^{ox} / V[a]	E^{red}/ V[a]	HOMO / eV[b]	LUMO / eV[b]
**Cz-C-PTZSO$_2$**	1.31	-2.32	-5.66	-2.03
**Cz-CO-PTZSO$_2$**	1.42	-1.52	-5.77	-2.83
**Cz-C-TRZ**	1.23	-1.73	-5.57	-2.62
**Cz-CO-TRZ**	1.41	-1.18	-5.76	-3.17

[a]Potential values were obtained from the DPV peak values in DMF and referenced with respect to SCE (Fc/Fc^+ = 0.45 eV).[8] [b]$E_{HOMO/LUMO}$= -(E^{ox}/E^{red} + 4.8) eV, using Fc/Fc^+ as the internal reference.[9]

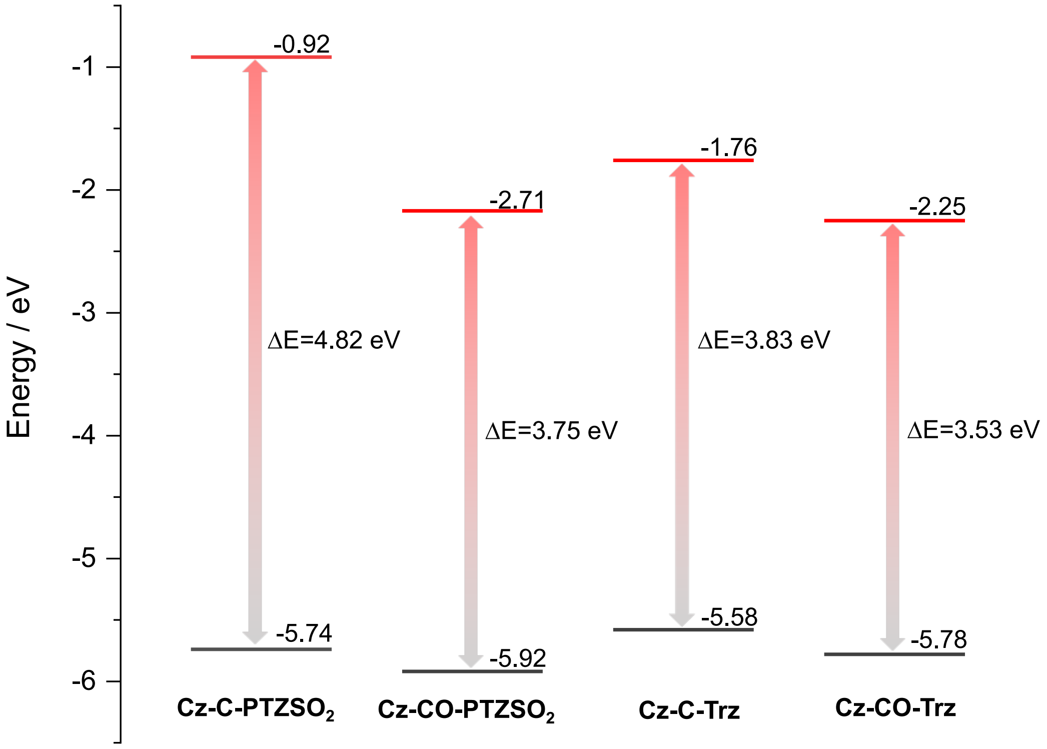

Figure S33. Calculated energies of the frontier molecular orbitals using the optimized S_0 geometry in the gas phase at the PBE0/6-31G(d,p) level.

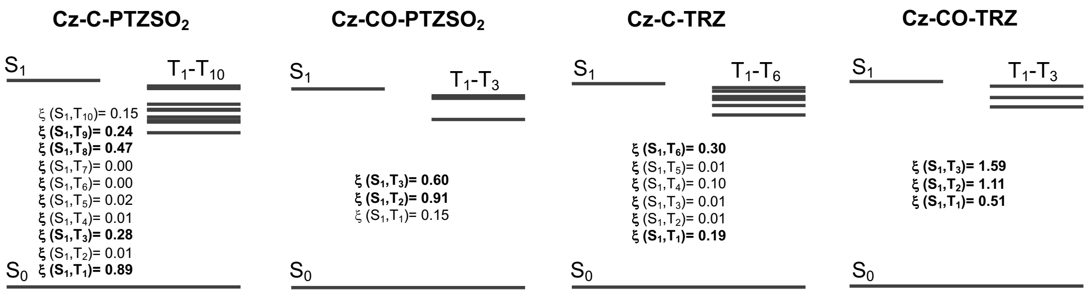

Figure S34. Vertical excitation energy levels calculated at the optimized S_0 geometry in the gas phase at TD-DFT-PBE0/6-31G(d,p) level; spin-orbit coupling constants calculated at the S_1 geometry.

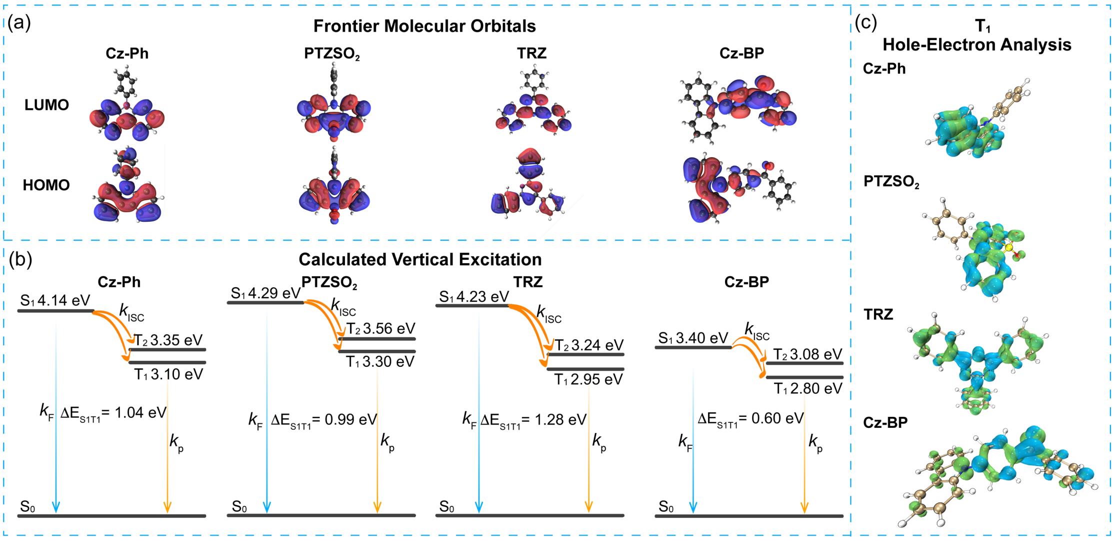

Figure S35. (a) Frontier molecular orbitals (isovalue: 0.02) and (b) vertical excitation energy levels of S_1, T_1 and T_2 states calculated at the optimized S_0 geometry in the gas phase at PBE0/6-31G(d,p) level. (c) Hole (green)-electron (blue) distribution analysis (isovalue: 0.002) for T_1 at the optimized S_0 geometry using Multiwfn program.[16]

Table S2. Summary of photophysical data of **Cz-C-PTZSO$_2$**, **Cz-CO-PTZSO$_2$**, **Cz-C-TRZ** and **Cz-CO-TRZ**.

	Fluorescence[a]		RTP[b]		LTP[c]		$\Phi_{PL}{}^{d}$ /%			
							Air		N_2	
	λ_{PL} /nm	τ_{PL} /ns	λ_{PL} /nm	τ_{PL} /ms	λ_{PL} /nm	τ_{PL} /ms	$\Phi_{PL}{}^{d}$	$\Phi_{P}{}^{e}$	Φ_{PL}	Φ_{P}
**Cz-C-PTZSO$_2$**	403<br>426	3.4<br>3.5	470<br>530	60.1<br>89.6	470<br>530	837.6<br>537.6	5.9	1.6	8.2	3.9
**Cz-CO-PTZSO$_2$**	440	0.7	520<br>572	12.3<br>244.3	484<br>512	84.2<br>89.8	4.6	0.0<br>2	4.7	0.1
**Cz-C-TRZ**	448	5.1	462<br>576	7.1<br>197.0	515	400.0	6.3	0.3	6.6	0.6
**Cz-CO-TRZ**	468	2.4	--	--	465<br>496<br>532<br>574	33.6<br>33.4<br>31.3<br>264.3	13.9	--	14.0	--

[a] Fluorescence in air at 298 K. [b] Phosphorescence in vacuum at 298 K. [c] Phosphorescence in vacuum at 80 K. [d] Total photoluminescence quantum yield. [e] Photoluminescence quantum yield of phosphorescence in the crystal state (λ_{exc} = 370 nm); RTP PLQYs were integrated from the phosphorescence emission band.

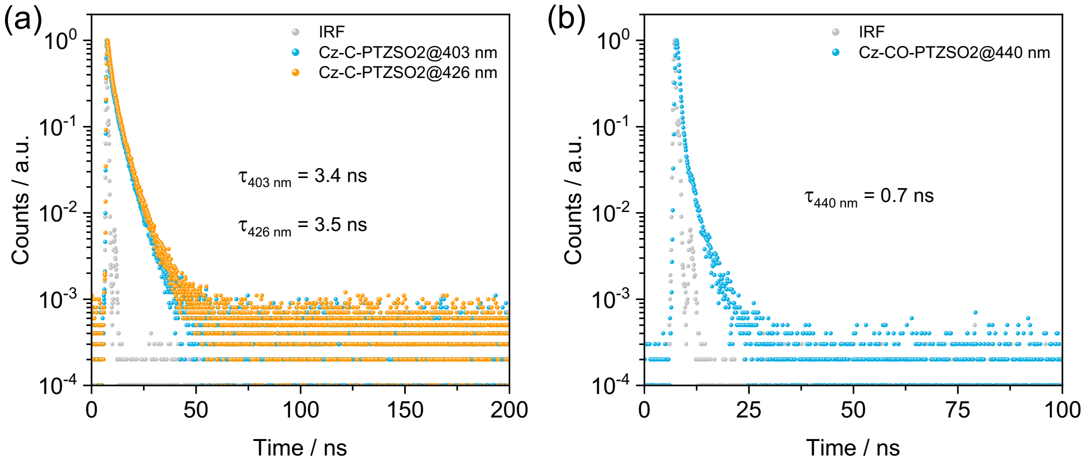

Figure S36. Time-resolved PL decay profile of (a) **Cz-C-PTZSO$_2$** and (b) **Cz-CO-PTZSO$_2$** in air at 298 K.

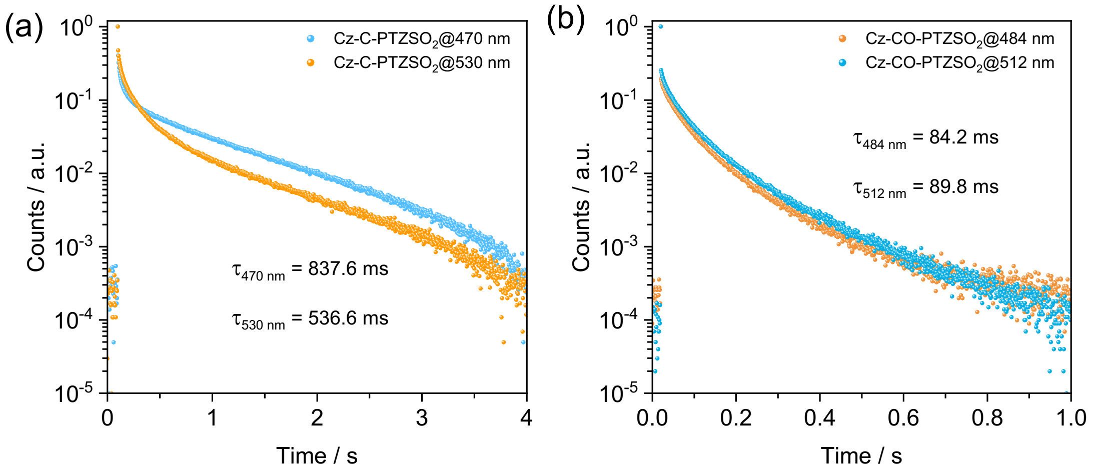

Figure S37. Time-resolved phosphorescence decay profile of (a) **Cz-C-PTZSO$_2$** and (b) **Cz-CO-PTZSO$_2$** in vacuum at 80 K.

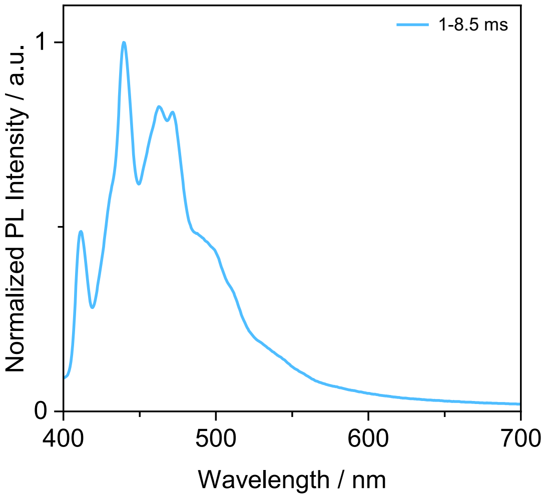

Figure S38. Phosphorescence spectra of **Cz-Ph** in 2-MeTHF at 77 K; time-gated window: 1-8.5 ms (λ_{exc} = 343 nm); concentration: 5×10^{-6} M.

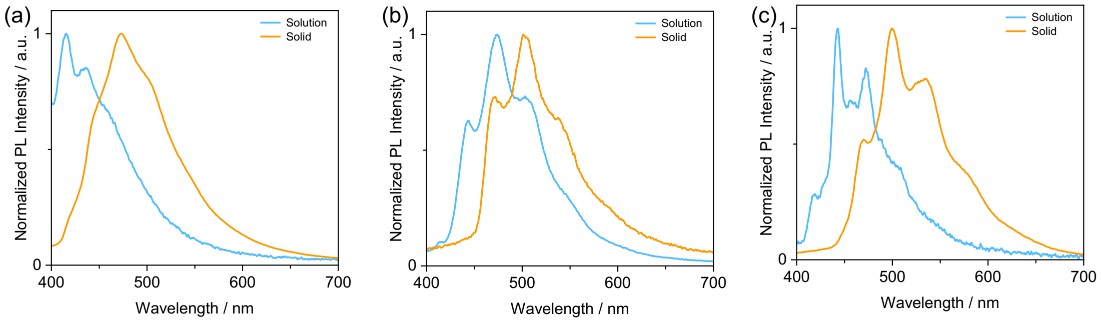

Figure S39. Phosphorescence spectra of (a) **PTZSO$_2$** and (b) **Cz-BP** and (c) **TRZ** in 2-MeTHF and in aggregates at 77 K; time-gated window: 1-8.5 ms (λ_{exc} = 343 nm); solution concentration: 5×10^{-6} M.

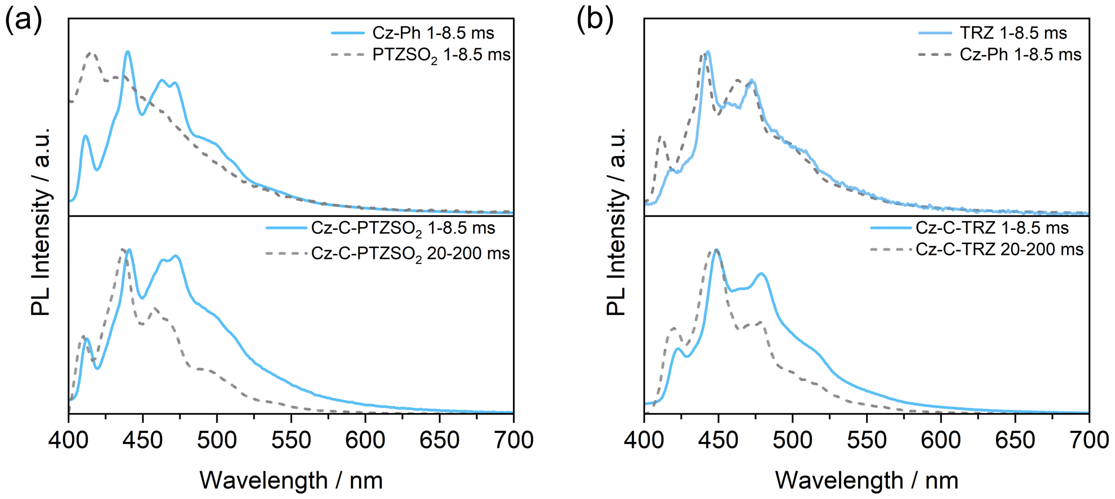

Figure S40. Comparison of phosphorescence spectra of (a) **Cz-C-PTZSO$_2$**, **Cz-Ph** and **PTZSO$_2$**, and (b) **Cz-C-TRZ**, **Cz-Ph** and **TRZ** in 2-MeTHF at 77 K (λ_{exc} = 343 nm); concentration: 5×10^{-6} M. Time-gated windows are shown in the figures.

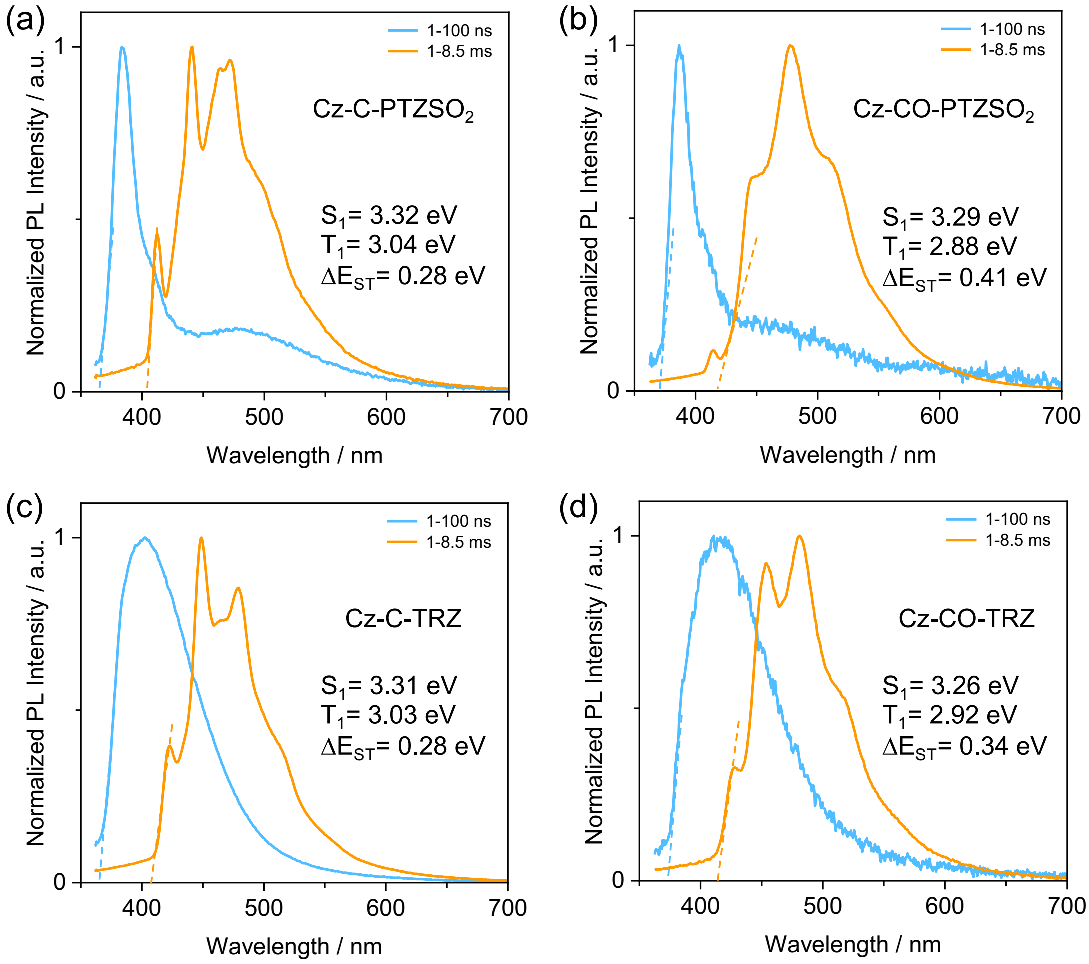

Figure S41. Prompt (1-100 ns) and delayed (1-8.5 ms) emission spectra of (a) **Cz-C-PTZSO$_2$**, (b) **Cz-CO-PTZSO$_2$**, (c) **Cz-C-TRZ** and (d) **Cz-CO-TRZ** in 2-MeTHF at 77 K (λ_{exc} = 343 nm); concentration: 5×10^{-6} M.

Table S3. Summary of calculated rate constants.

	Φ_p / % [a]	Φ_d / % [b]	k_p / s^{-1} [c]	k_r^S / s^{-1} [d]	k_d / s^{-1} [e]	k_r^T / s^{-1} [f]	k_{ISC} / s^{-1} [g]	k_{nr}^T / s^{-1} [h]
**Cz-C-PTZSO$_2$**	4.3	3.9	2.94×10^8	1.26×10^7	1.66×10^1 (T_1^H) [e1]<br>1.12×10^1 (T_1^L) [e2]	6.78×10^{-1} (T_1^H) [f1]<br>4.55×10^{-1} (T_1^L) [f2]	2.81×10^8	1.59×10^1 (T_1^H) [h1]<br>1.07×10^1 (T_1^L) [h2]
**Cz-CO-PTZSO$_2$**	4.6	0.1	1.43×10^9	6.57×10^7	8.13×10^1 (T_1) [e3]<br>4.09 (T_1^*) [e4]	8.52×10^{-2} (T_1) [f3]<br>4.29×10^{-3} (T_1^*) [f4]	1.36×10^9	8.12×10^1 (T_1) [h3]<br>4.08 (T_1^*) [h4]
**Cz-C-TRZ**	6.0	0.6	1.96×10^8	1.18×10^7	1.41×10^2 (T_1^H) [e1]<br>5.08 (T_1^L) [e2]	8.99×10^{-1} (T_1^H) [f1]<br>3.24×10^{-2} (T_1^L) [f2]	1.84×10^8	1.40×10^2 (T_1^H) [h1]<br>5.05 (T_1^L) [h2]
**Cz-CO-TRZ**	13.9	--	4.17×10^8	5.79×10^7	--	--	3.59×10^8	--

[a] Φ_p recorded in air. [b] Φ_d recorded in N_2. [c] Prompt fluorescence decay rate. [d] Intrinsic S_1 decay rate; [e] RTP decay rate; [e1] Measured T_1^H phosphorescence decay rate; [e2] Measured T_1^L phosphorescence decay rate; [e3] Measured T_1 phosphorescence decay rate (monomer); [e4] Measured T_1 phosphorescence decay rate (aggregate); [f] Intrinsic decay rate of the triplet excited state; [f1] Intrinsic T_1^H phosphorescence decay rate; [f2] Intrinsic T_1^L phosphorescence decay rate; [f3] Intrinsic T_1 phosphorescence decay rate (monomer); [f4] Intrinsic T_1 phosphorescence decay rate (aggregate); [g] Average intersystem crossing rate; [h] Non-radiative decay rate of the triplet excited state; [h1] Non-radiative T_1^H decay rate; [h2] Non-radiative T_1^L decay rate; [h3] Non-radiative T_1 decay rate (monomer); [h4] Non-radiative T_1 decay rate (aggregate). No phosphorescence was detected in **Cz-CO-TRZ**.

The related calculation equations for the different rates were deduced based on our previous work (*J. Phys. Chem. A*, **2021**, *125*, 8074-8089) and informed by others (*Nat. Rev. Mater.* **2020**, *5*, 869–885; *Acc. Chem. Res.* **2021**, *54*, 940–949), as follows:

$$\Phi_p = \frac{k_r^S}{k_r^S+k_{ISC}+k_{nr}^S} \qquad \text{eq. 1}$$

$$\tau_p = \frac{1}{k_r^S+k_{ISC}+k_{nr}^S} \qquad \text{eq. 2}$$

$$k_p = k_r^S + k_{ISC} + k_{nr}^S \qquad \text{eq. 3}$$

$$k_d = k_{nr}^T + k_r^T \qquad \text{eq. 4}$$

$$\tau_d = \frac{1}{k_r^T+k_{nr}^T} \qquad \text{eq. 5}$$

$$\Phi_d = \Phi_{ISC} k_r^T \tau_d \qquad \text{eq. 6}$$

Assuming $k_{nr}^S \approx 0$, the following equations can be deduced based on eqs. 1-6:

$$k_r^S = k_p \Phi_p \qquad \text{eq. 7}$$

$$k_{ISC} = k_p(1 - \Phi_p) \qquad \text{eq. 8}$$

$$\Phi_{ISC} = \frac{k_{ISC}}{k_r^S+k_{ISC}} \qquad \text{eq. 9}$$

$$k_r^T = \frac{\Phi_d}{\Phi_{ISC}\tau_d} \qquad \text{eq. 10}$$

$$k_{nr}^T = k_d - k_r^T \qquad \text{eq. 11}$$

Based on the above eqs.7-11, the calculated rate constants are presented in Table **S3**.

Note:

1) k_{nr}^S was assumed to be 0.
2) Φ_d and Φ_{ISC} were used for calculating k_{ISC} without differentiating the contribution from two triplet excited states to the quantum efficiencies, restricted by the instrumentation.

Although the value of these data is limited by the range of values each rate constant can adopt based on the inherent assumptions made, they can be used as a qualitative analysis for the IC process. From the k_{nr}^T column of Table **S3**, relatively large k_{nr}^T values for the T_1 state were calculated. As small RTP F_{PL} (F_d) values indicate, fast non-radiative transitions occur in these two compounds. **Cz-C-PTZSO$_2$** and **Cz-C-TRZ** possess relatively large k_{nr}^T values from the T_1^H states. Considering the much larger energy gap between T_1^H and S_0 compared to that between T_1^H and T_1^L states, the IC from T_1^H to T_1^L states can be considered as the dominant non-radiative pathway from the T_2 state. Thus, k_r^T (T_1^H) is able to compete with the IC (from T_1^H and T_1 states) for T_2 phosphorescence to be observable. The low F_d values result from relatively larger k_{nr}^T values compared to k_r^T values.

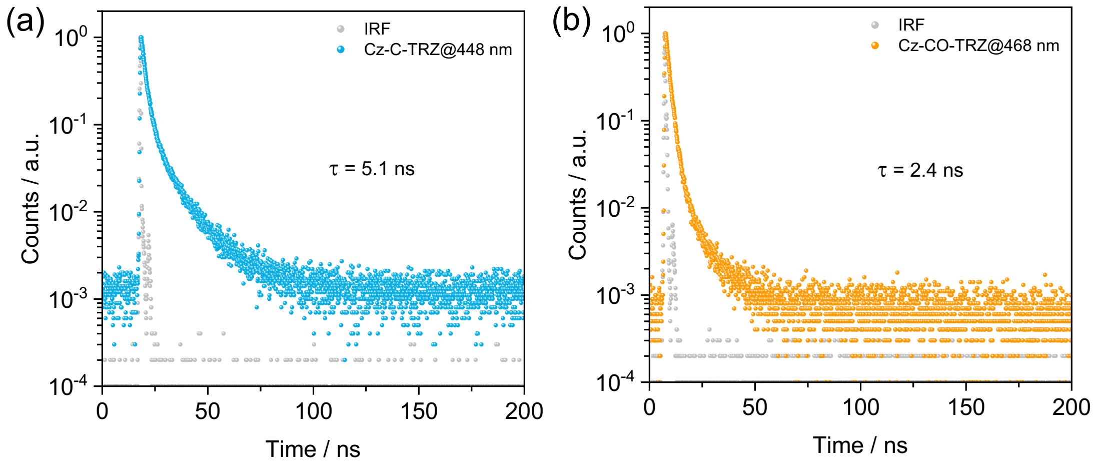

Figure S42. Time-resolved PL decay profile of (a) **Cz-C-TRZ** and (b) **Cz-CO-TRZ** in air at 298 K.

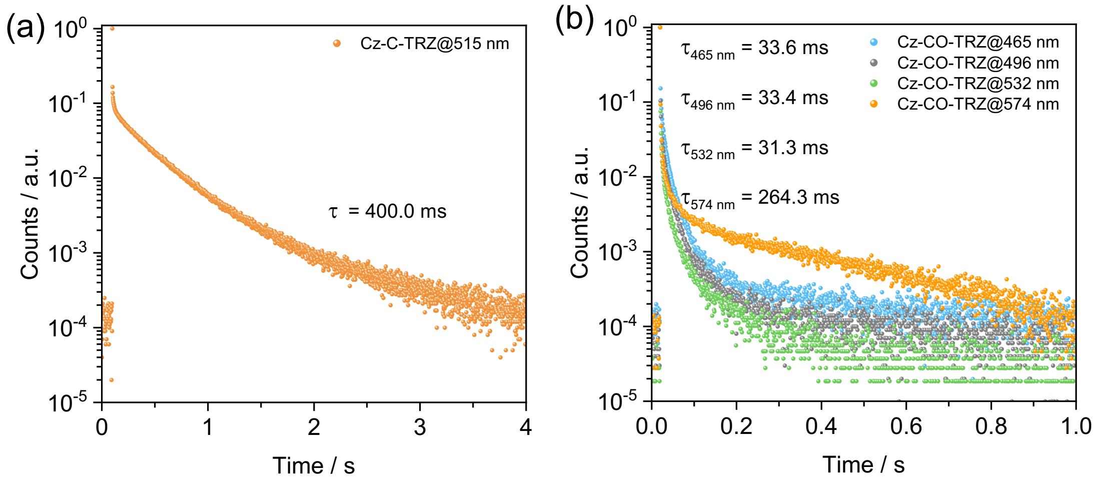

Figure S43. Time-resolved phosphorescence decay profile of (a) **Cz-C-TRZ** and (b) **Cz-CO-TRZ** in vacuum at 80 K.

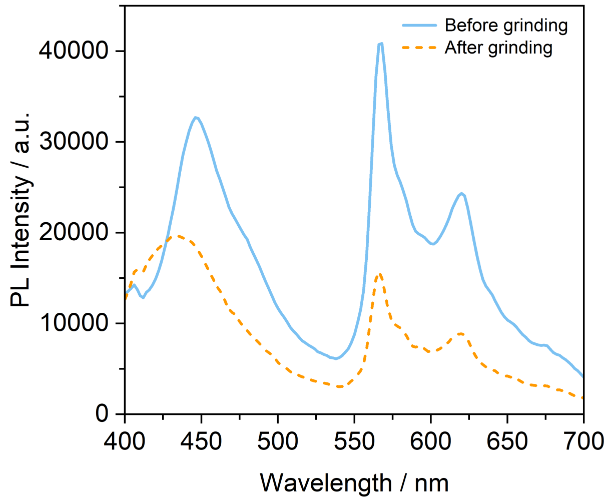

Figure S44. RTP spectra of **Cz-C-TRZ** crystals isolated from toluene before and after grinding (time-gated window: 0.05-1 s).

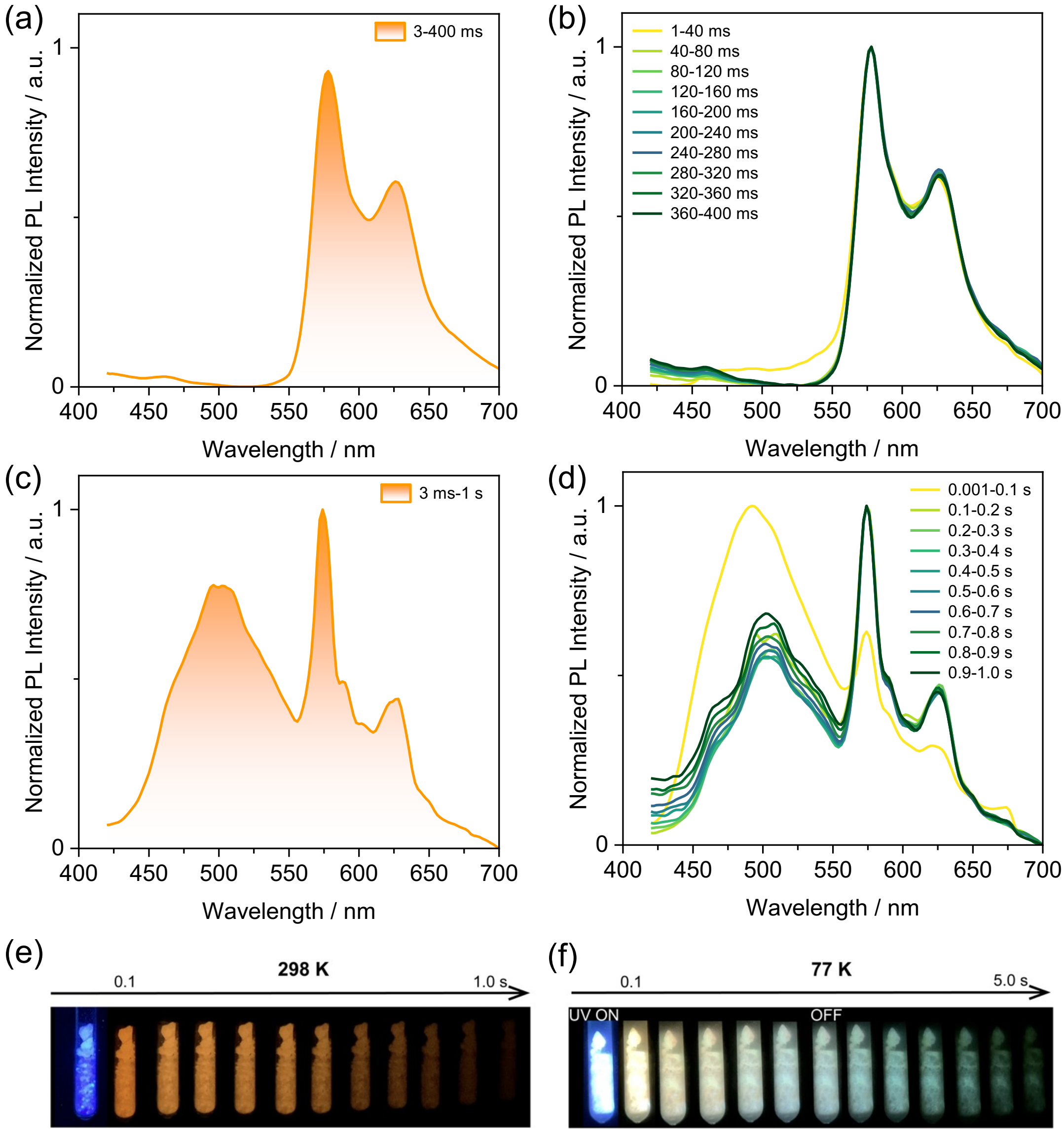

Figure S45. (a) RTP spectrum of closely stacked **Cz-C-TRZ** crystal at 298 K. (b) RTP spectra of closely **Cz-C-TRZ** crystal recorded at different time-gated windows. (c) LTP spectrum of closely **Cz-C-TRZ** crystal at 80 K. (d) LTP of closely **Cz-C-TRZ** crystal recorded at different time-gated windows. Images showing the phosphorescence afterglows at (e) 298 K and (f) 77 K. The closely stacked **Cz-C-TRZ** crystal was isolated following vacuum sublimation.

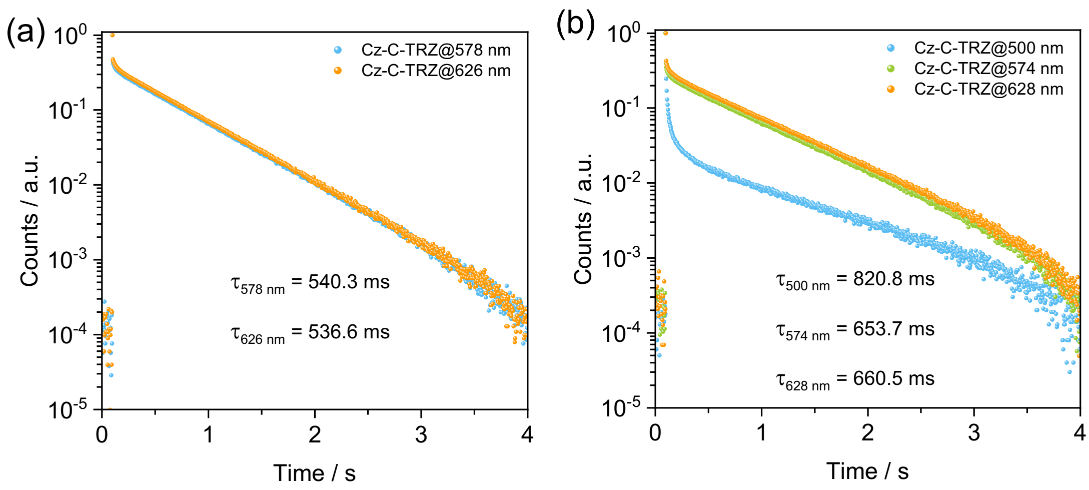

Figure S46. Time-resolved phosphorescence decay profiles of closely stacked **Cz-C-TRZ** crystal at (a) 298 K and (b) 80 K.

Table S4. Summary of photophysical data of the closely stacked **Cz-C-TRZ** crystals.

	RTP[a]		LTP[b]		Φ_{PL}[c] /%			
					Air		N_2	
	λ_{PL} /nm	τ_{PL} /ms	λ_{PL} /nm	τ_{PL} /ms	Φ_{PL}[c]	Φ_{P}[d]	Φ_{PL}	Φ_{P}
**Cz-C-TRZ**	578<br>626	540.3<br>536.6	500<br>574<br>628	820.8<br>653.7<br>660.5	9.2	2.8	9.4	3.0

[a] Phosphorescence in vacuum at 298 K. [b] Phosphorescence in vacuum at 80 K. [c] Total photoluminescence quantum yield. [d] Photoluminescence quantum yield of phosphorescence (λ_{exc} = 370 nm); RTP PLQYs were integrated from the phosphorescence emission band.

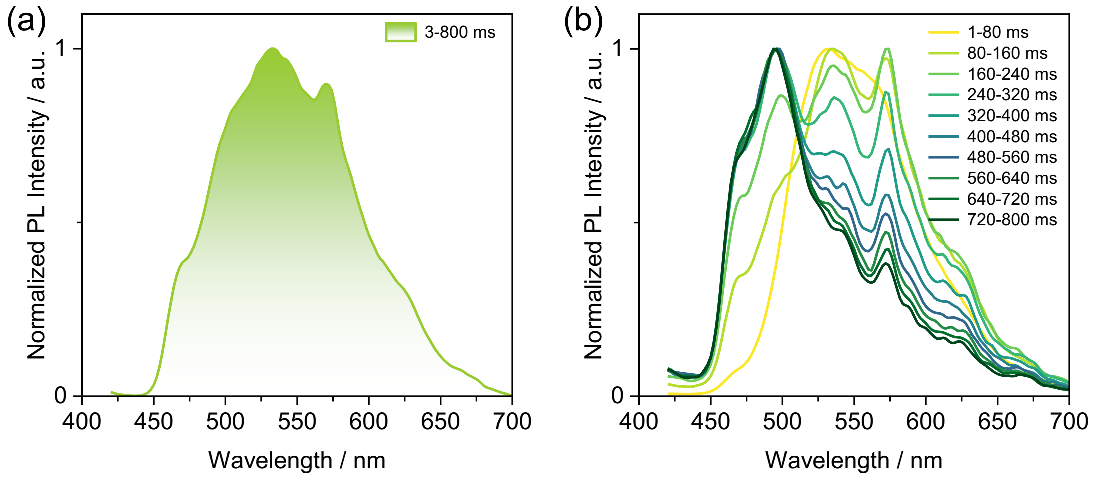

Figure S47. (a) LTP spectrum of **Cz-CO-TRZ** crystal at 80 K. (b) LTP of **Cz-CO-TRZ** recorded at different time-gated windows.

References

(1) Gomurashvili, Z.; Crivello, J. V. Monomeric and polymeric phenothiazine photosensitizers for photoinitiated cationic polymerization. *Macromolecules* **2002**, *35* (8), 2962-2969.
(2) Expert, C.-S. V2. 1. *Rigaku Americas: The Woodlands, TX, USA* **2015**.
(3) Greenham, N.; Samuel, I.; Hayes, G.; Phillips, R.; Kessener, Y.; Moratti, S.; Holmes, A.; Friend, R. Measurement of absolute photoluminescence quantum efficiencies in conjugated polymers. *Chem. Phys. Lett.* **1995**, *241* (1-2), 89-96.
(4) CrysAlisPRO, O. D. Agilent technologies uk ltd. *Yarnton, England* **2014**, *1* (39.34).
(5) Expert, C.-S. Rigaku corporation. *Tokyo, Japan* **2011**.
(6) Sheldrick, G. M. Shelxt–integrated space-group and crystal-structure determination. *Acta Crystallographica Section A: Foundations and Advances* **2015**, *71* (1), 3-8.
(7) Dolomanov, O. V.; Bourhis, L. J.; Gildea, R. J.; Howard, J. A.; Puschmann, H. Olex2: A complete structure solution, refinement and analysis program. *J. Appl. Crystallogr.* **2009**, *42* (2), 339-341.
(8) Connelly, N. G.; Geiger, W. E. Chemical redox agents for organometallic chemistry. *Chem. Rev.* **1996**, *96* (2), 877-910.
(9) Cardona, C. M.; Li, W.; Kaifer, A. E.; Stockdale, D.; Bazan, G. C. Electrochemical considerations for determining absolute frontier orbital energy levels of conjugated polymers for solar cell applications. *Adv. Mater.* **2011**, *23* (20), 2367-2371.
(10) *Gaussian 16 rev. C.01*; Wallingford, CT, 2016.
(11) Adamo, C.; Barone, V. Toward reliable density functional methods without adjustable parameters: The pbe0 model. *J. Chem. Phys.* **1999**, *110* (13), 6158-6170.
(12) Petersson, G.; Tensfeldt, T. G.; Montgomery Jr, J. A complete basis set model chemistry. Iii. The complete basis set ‐ quadratic configuration interaction family of methods. *J. Chem. Phys.* **1991**, *94* (9), 6091-6101.
(13) Gao, X.; Bai, S.; Fazzi, D.; Niehaus, T.; Barbatti, M.; Thiel, W. Evaluation of spin-orbit couplings with linear-response time-dependent density functional methods. *J. Chem. Theory Comput.* **2017**, *13* (2), 515-524.
(14) Dennington, R.; Keith, T. A.; Millam, J. M. Gaussview 6.0. 16. *Semichem Inc.: Shawnee Mission, KS, USA* **2016**.
(15) Johnson, E. R.; Keinan, S.; Mori-Sánchez, P.; Contreras-García, J.; Cohen, A. J.; Yang, W. Revealing noncovalent interactions. *J. Am. Chem. Soc.* **2010**, *132* (18), 6498-6506.
(16) Lu, T.; Chen, F. Multiwfn: A multifunctional wavefunction analyzer. *J. Comput. Chem.* **2012**, *33* (5), 580-592.
(17) Humphrey, W.; Dalke, A.; Schulten, K. Vmd: Visual molecular dynamics. *J. Mol. Graphics* **1996**, *14* (1), 33-38.
(18) Niu, Z.; Wu, H.; Liu, L.; Dai, G.; Xiong, S.; Zhao, Y.; Zhang, X. Chain rigidity modification to promote the electrochemical performance of polymeric battery electrode materials. *J. Mater. Chem. A* **2019**, *7* (17), 10581-10588.
(19) Esmaeilpour, M.; Javidi, J. Magnetically-recoverable schiff base complex of pd (ii) immobilized on fe3o4@sio2 nanoparticles: An efficient catalyst for mizoroki-heck and suzuki-miyaura coupling reactions. *J. Chin. Chem. Soc.* **2015**, *62* (7), 614-626.

(20) Mao, M.; Ren, M.-G.; Song, Q.-H. Thermodynamics and conformations in the formation of excited states and their interconversions for twisted donor-substituted tridurylboranes. *Chem. Eur. J.* **2012**, *18* (48), 15512-15522.
(21) Tian, Y.; Yang, J.; Liu, Z.; Gao, M.; Li, X.; Che, W.; Fang, M.; Li, Z. Multistage stimulus-responsive room temperature phosphorescence based on host–guest doping systems. *Angew. Chem. Int. Ed.* **2021**, *60* (37), 20259-20263.
(22) Shi, H.; Gong, Z.; Xin, D.; Roose, J.; Peng, H.; Chen, S.; Lam, J. W. Y.; Tang, B. Z. Synthesis, aggregation-induced emission and electroluminescence properties of a novel compound containing tetraphenylethene, carbazole and dimesitylboron moieties. *J. Mater. Chem. C* **2015**, *3* (35), 9095-9102.
(23) Ma, F.; Ji, H.; Zhang, D.; Xue, K.; Zhang, P.; Qi, Z.; Zhu, H. Adjusting the photophysical properties of aie-active tadf emitters from through-bond to through-space charge transfer for high-performance solution-processed oleds. *Dyes Pigments* **2021**, *188*, 109208.
(24) Matsuo, Y.; Wang, Y.; Ueno, H.; Nakagawa, T.; Okada, H. Mechanochromism, twisted/folded structure determination, and derivatization of (n-phenylfluorenylidene)acridane. *Angew. Chem. Int. Ed.* **2019**, *58* (26), 8762-8767.
(25) Ishiyama, T.; Murata, M.; Miyaura, N. Palladium (0)-catalyzed cross-coupling reaction of alkoxydiboron with haloarenes: A direct procedure for arylboronic esters. *J. Org. Chem.* **1995**, *60* (23), 7508-7510.
(26) Wang, T.; Hu, Z.; Nie, X.; Huang, L.; Hui, M.; Sun, X.; Zhang, G. Thermochromic aggregation-induced dual phosphorescence via temperature-dependent sp3-linked donor-acceptor electronic coupling. *Nat. Commun.* **2021**, *12* (1), 1364.
